# Multi-way metamodelling facilitates insight into the complex input-output maps of nonlinear dynamic models

**DOI:** 10.1186/1752-0509-6-88

**Published:** 2012-07-20

**Authors:** Kristin Tøndel, Ulf G Indahl, Arne B Gjuvsland, Stig W Omholt, Harald Martens

**Affiliations:** 1 Centre for Integrative Genetics (CIGENE), Dept. of Mathematical Sciences and Technology, Norwegian University of Life Sciences, P. O. Box 5003, N-1432, Ås, Norway; 2 CIGENE, Dept. of Animal and Aquacultural Sciences, Norwegian University of Life Sciences, P. O. Box 5003, N-1432, Ås, Norway; 3 Nofima, P. O. Box 210, N-1431, Ås, Norway

**Keywords:** Parameter-phenotype map, Dynamic models, Metamodelling, N-way Partial Least Squares Regression, Hierarchical analysis, HC-PLSR, Cluster-analysis, Input-output relationships, Circadian clock

## Abstract

**Background:**

Statistical approaches to describing the behaviour, including the complex relationships between input parameters and model outputs, of nonlinear dynamic models (referred to as metamodelling) are gaining more and more acceptance as a means for sensitivity analysis and to reduce computational demand. Understanding such input-output maps is necessary for efficient model construction and validation. Multi-way metamodelling provides the opportunity to retain the block-wise structure of the temporal data typically generated by dynamic models throughout the analysis. Furthermore, a cluster-based approach to regional metamodelling allows description of highly nonlinear input-output relationships, revealing additional patterns of covariation.

**Results:**

By presenting the N-way Hierarchical Cluster-based Partial Least Squares Regression (N-way HC-PLSR) method, we here combine multi-way analysis with regional cluster-based metamodelling, together making a powerful methodology for extensive exploration of the input-output maps of complex dynamic models. We illustrate the potential of the N-way HC-PLSR by applying it both to predict model outputs as functions of the input parameters, and in the inverse direction (predicting input parameters from the model outputs), to analyse the behaviour of a dynamic model of the mammalian circadian clock. Our results display a more complete cartography of how variation in input parameters is reflected in the temporal behaviour of multiple model outputs than has been previously reported.

**Conclusions:**

Our results indicated that the N-way HC-PLSR metamodelling provides a gain in insight into which parameters that are related to a specific model output behaviour, as well as variations in the model sensitivity to certain input parameters across the model output space. Moreover, the N-way approach allows a more transparent and detailed exploration of the temporal dimension of complex dynamic models, compared to alternative 2-way methods.

## Background

Dynamic models in systems biology as well as in other fields become increasingly complex as more detailed knowledge is incorporated. The massive presence of nonlinear relationships between their high-dimensional parameter- and solution- spaces is a key characteristic of such systems. Moreover, dynamic models typically generate multidimensional blocks of temporal data. Clearly it is very challenging to obtain a comprehensive overview of the behavioural repertoires of such models across the high-dimensional input parameter space, including the sensitivity of the model output to changes in the various input parameters, as well as interactions between input parameters and correlation patterns between model outputs. For dynamic model construction and validation, sound handling of such information is crucial. Since most of the existing methods for parameter estimation and sensitivity analysis are appropriate only for systems of relatively low output dimensionality and typically focus on one output variable at a time 1,2, a generic methodology for analysis of model behaviour that is able to handle the entire range of model complexities and give a comprehensive overview of the relationships between the input parameters and all model outputs, is sorely needed.

Statistical approaches are gaining acceptance as a means for analysis of input-output relationships of complex dynamic models 2,3,4,5,6,7,8,9,10, and statistical emulation of dynamic models (metamodelling 11) has been demonstrated to be a useful tool both for speeding up computations 12 and as a basis for sensitivity analysis 2,3,13 and uncertainty assessment 14,15,16. Multi-way (N-way) methods have previously been shown to be effective for data integration in e.g. systems biology 17,18. We therefore hypothesise that N-way approaches will be especially advantageous for metamodelling of dynamic models due to the capability of integrating temporal data from several output state variables simultaneously while retaining the information about which state trajectory that corresponds to which state variable throughout the analysis (with 2-way methods, this information is lost when concatenating the trajectories for the different state variables prior to the analysis). Consequently, a more detailed exploration of the temporal dimension of dynamic models is possible. This is important in order to obtain a comprehensive overview of how variation in the input parameters is manifested in the model output. Moreover, methods utilising several model outputs simultaneously have already been demonstrated to reduce the model sloppiness by imposing more constraints on the system 5.

**Figure 1:**
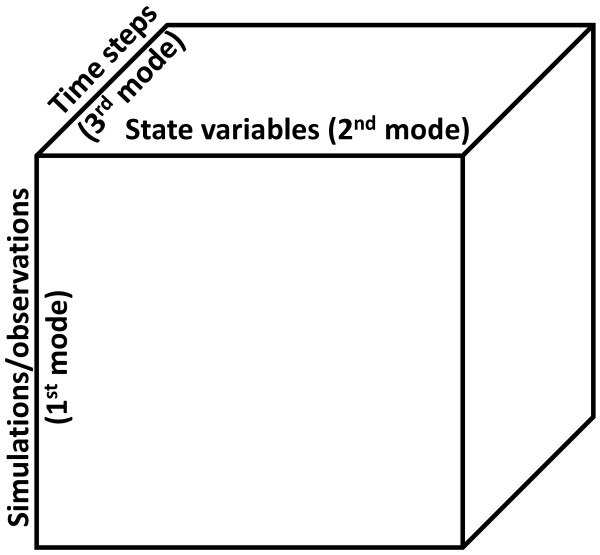
**Illustration of the N-way data structure used in N-way HC-PLSR. **Illustration of the data structure used in N-way metamodelling. Here the number of modes (ways) N = 3, where the first mode is the different simulations carried out using varying parameter combinations and/or initial conditions, the second mode is the various state variables of the analysed dynamic model and the third mode is the trajectories of the state variables. Hence, the data is here represented as a 3-way array. However, using more than three modes is possible. The decomposition of the 3-way data is described and illustrated in Additional file 1: Section S1.

The N-way Hierarchical Cluster-based Partial Least Squares Regression (N-way HC-PLSR) presented here is designed for efficient handling of block-wise *nonlinear* data structures, and works by combining several regional N-way Partial Least Squares Regression (NPLSR) 19 models within which the mappings between input parameters and output state variables can be more adequately represented than in a global NPLSR model. The nonlinear capabilities of this metamodelling approach are obtained by combining clustering and generation of local linear metamodels for the various cluster regions. This is an N-way extension of our previously published method Hierarchical Cluster-based Partial Least Squares Regression (HC-PLSR) 8. HC-PLSR is based on separate (2-way) PLSR 20,21,22,23 analyses of distinct regions of the parameter space (where the resulting regression coefficients are measures of the model sensitivity to the different input parameters), while in N-way HC-PLSR, the separated regions are defined according to the dynamic behaviour of the output state variables and the output from the dynamic model is represented as an N-way array (in our example the number of ways or modes N=3; observations×state variables×time points of the state trajectories (see Figure 1)). A common metamodel based on all state variable trajectories can thereby be generated. This allows simultaneous analysis of nonlinear relationships between all model outputs and input parameters of complex dynamic models, in a low-dimensional subspace spanned by estimated latent variables (called NPLSR factors). The NPLSR factors represent the features that are most important for the covariance between the inputs and outputs (see Additional file 1: Section S1 for a description of the NPLSR methodology). The method is therefore suited for visualising covariance structures both within and between the input parameters and the model outputs, and thereby also useful for finding and removing possible redundancies (leading to model reduction) and prioritizing experiments for e.g. model validation by identification of key inputs and outputs describing the system behaviour. Hence, this can also guide experimentalists in the choice of quantities to measure when studying a biological system. Moreover, the NPLSR approach provides considerable dimension reduction possibilities through projection of the data into a low-dimensional subspace. In our opinion, this methodology should be considered as particularly useful in future multivariate metamodelling for analysis of complex spatiotemporal models. In N-way metamodelling, the three spatial dimensions can be included as separate modes in the N-way analysis, in addition to the temporal dimension on which we focus in this paper. The spatial structure of the data can thereby be kept throughout the analysis. We hypothesise that this will be a great advantage in the analysis of spatiotemporal models.

Traditionally, metamodelling is carried out in the causal direction, predicting model outputs as functions of the input parameters using e.g. regression methods. Application of metamodelling in the reverse direction is, however, also of potential interest 5. The two modelling directions can be understood as extensions of the classical/inverse calibration modelling 22. Accordingly, we refer to the causal direction as *classical metamodelling*, and the reverse direction as *inverse metamodelling*, and in our application of the N-way HC-PLSR we demonstrate how their combination provides more detailed insight into the complexity of the mapping between input parameters and model outputs. Inverse metamodelling may also facilitate fitting of nonlinear models to large amounts of experimental data. Given that the results from the computations can be substituted with relevant experimentally measured data or quantities calculated from measurements, these can be used to predict corresponding parameters. Moreover, combinations of classical and inverse metamodelling can identify the key metrics to measure in order to validate the models. A more comprehensive introduction to the multivariate metamodelling methodology is given in Additional file 1: Section S1.

As long as they handle high-dimensional data with nonlinear relationships and yield interpretable representations, a wide variety of statistical methods can be effectively used for multivariate metamodelling. We have recently shown that multivariate metamodelling based on PLSR and our nonlinear extension HC-PLSR 8 provides good approximations of the input-output mappings 8 as well as informative insight into complex interaction patterns between parameters 9 of advanced nonlinear dynamic models. PLSR can use multiple response variables simultaneously and utilise inter-correlations between them for model stabilisation. PLSR analysis has been shown to effectively reveal covariation patterns in large and complex data sets, and extract correlations between possibly noisy and partially redundant input variables and outputs 6. The success of PLSR in the context of sensitivity analysis and for constraining input parameter values from dynamic model outputs has also been demonstrated by Sobie et al. 5,6. Highly nonlinear input-output structures may, however, be difficult to model adequately with linear models such as PLSR, even with polynomial extensions. To confront these problems, HC-PLSR was introduced 8. Heterogeneity in model sensitivity to certain parameters between various regions in the parameter space of a dynamic model of the mouse ventricular myocyte was identified by HC-PLSR-based sensitivity analysis in 9. Similarly, zooming into different regions of the state variable behavioural domain provides the opportunity to identify regions where the relationship between certain parameters and the model output is less ambiguous, indicating that these parameters are especially important for defining a specific type of temporal model behaviour. In cases where variation in the input parameters can be directly related to genotypic variations, this may provide valuable information about how a specific genotype can be of particular importance for the manifestation of certain phenotypic characteristics.

Here, we combine three different aspects of multivariate metamodelling: 1) Description of highly nonlinear input-output relationships by regional metamodelling, 2) NPLSR, allowing a retention of a tensor data structure throughout the analysis and 3) Inverse metamodelling in addition to the classical approach, providing more confident conclusions and a more comprehensive model overview. Moreover, particularly complex details are pursued by more detailed metamodelling of individual outputs and their relationships to the varied input parameters. Altogether, this provides a powerful, robust and efficient approach to exploration of the behavioural repertoire of complex dynamic models.

We illustrate our methodology by an application to a complex dynamic model of the mammalian circadian clock developed by Leloup and Goldbeter 24, which is a well-established and validated model. Models of circadian rhythms have e.g. been used for identifying mechanisms of chronotolerance and chronoefficacy for anticancer drugs 25. The dynamic model we analyse describes circadian oscillations of cellular activity in conditions of continuous darkness, and consists of 16 coupled ordinary differential equations (ODEs) describing the dynamics of three genes through intertwined positive and negative feedback loops. By combining the classical and inverse approaches of the N-way HC-PLSR, we capture several interesting parts of the present complex input-output relationships, which are difficult to deduce directly from the model’s differential equations.

## Results

### *In silico* data set

The analysed mammalian circadian clock model consisted of 16 linear and nonlinear ODEs coupled together through numerous feedback mechanisms. To analyse the behaviour of this complex nonlinear dynamic model, nine of the model input parameters were systematically varied at eight equally spaced levels each in an Optimised Multi-level Binary Replacement (OMBR) design 7,26, using the ranges given in Table 1. This resulted in 8192 simulations with the circadian clock model, 99.3% of which (8135 simulations) converged to a stable limit cycle. The results of these 8135 simulations, represented as a 3-way array of 8135 observations x 16 state variables (corresponding to the 16 coupled differential equations in the dynamic model) x 200 time points in each trajectory, were related to the matrix of input parameter combinations (8135 x 9) in the metamodelling study described below.

**Table 1:** Description and range of the parameters varied in the mammalian circadian clock model simulations

**Parameter name**	**Unit**	**Description**	**Minimum value**	**Level step size**	**Maximum value**
*v*_*mB*_	nMh^-1^	Maximum rate of Bmal1 mRNA degradation	0.02	0.05	0.38
*v*_*mC*_	nMh^-1^	Maximum rate of Cry mRNA degradation	0.95	0.08	1.54
*v*_*mP*_	nMh^-1^	Maximum rate of Per mRNA degradation	0.98	0.16	2.09
*v*_*dPCN*_	nMh^-1^	Maximum rate of degradation of nuclear phosphorylated Per-Cry complex	0.99	0.02	1.14
*v*_*dIN*_	nMh^-1^	Maximum rate of degradation of nuclear Per-Cry-Clock-Bmal1 complex	0.08	0.21	1.52
*k*_*1*_	h^-1^	Rate constant for entry of the Per-Cry complex into the nucleus	0.08	0.21	1.52
*k*_*3*_	nM^-1^ h^-1^	Rate constant for the formation of the Per-Cry complex	0.08	0.21	1.52
*k*_*5*_	h^-1^	Rate constant for entry of the Bmal1 protein into the nucleus	0.27	0.02	0.41
*k*_*7*_	nM^-1^ h^-1^	Rate constant for the formation of the inactive Per-Cry-Clock-Bmal1 complex	0.05	0.13	0.95

A separate test set based on 8192 parameter combinations found by random Monte Carlo sampling 27,28 within the same parameter levels as used in the calibration set was also generated, resulting in 8125 converging simulations.

### Results from the N-way HC-PLSR metamodelling of the mammalian circadian clock model

**Figure 2:**
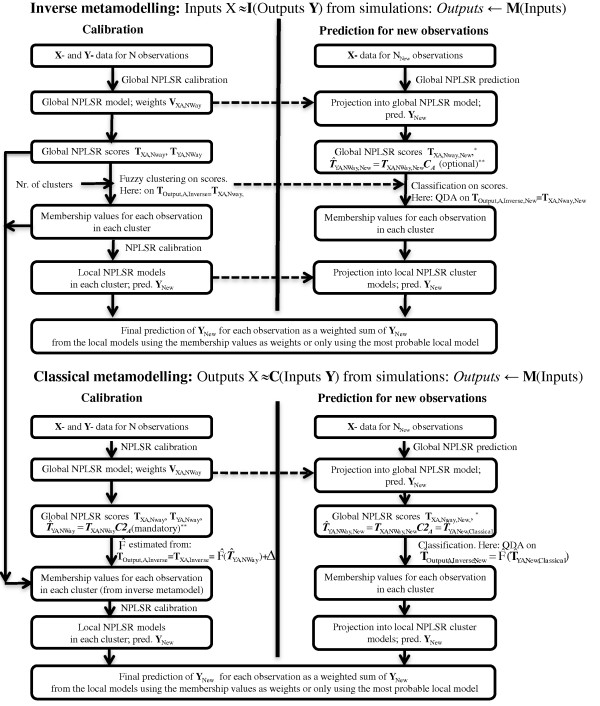
**Illustration of the combined classical and inverse N-way HC-PLSR metamodelling. **The inverse metamodelling was carried out first, defining the clusters to use also in the classical metamodelling. The classification of the test set observations to be predicted in the classical metamodelling was based on ${\hat{T}}_{\text{OutputA,Inverse}}$, predicted from ${\hat{T}}_{\text{YA,NWay}}$(see Additional file 1, eq. S12c for a definition) using second order polynomial Ordinary Least Squares (OLS) regression (called function $\hat{F}$*(.)*). See Additional file 1 sections S1.5-S1.7 for a more comprehensive description of this methodology, including predicting equations for test set observations. *See Additional file 1, equation S9b. ^**^C_***A ***_and ***C2***_***A ***_were calculated by equation S12b in Additional file 1.

A combined classical (parameter matrix as ***X***, 3-way state trajectory array as ***Y***) and inverse (3-way state trajectory array as ***X***, parameter matrix as ***Y***) metamodelling with N-way HC-PLSR was carried out, in order to assess the complex input-output map of the mammalian circadian clock model. The developed methodology is illustrated in Figure 2 and described in more detail in the Methods section and in Additional file 1: Section S1. The N-way HC-PLSR metamodels each contain both a global NPLSR model, and several regional NPLSR models calibrated within clusters corresponding to different model behaviour. Here we present the results from the global NPLSR metamodelling first, and the additional insights provided through the regional metamodelling thereafter. The statistics of the global metamodels can be found in Additional file 1: Section S2.

The low percentage explained ***Y****-*variance (Additional file 1: Section S2, Figure S3) showed that the global metamodelling was inadequate in both the classical and inverse direction. However, before proceeding to improved, regional metamodelling, the dominating relationships and patterns between the 9 input parameters and the 16 output state trajectories of the mammalian circadian clock model were assessed using the global approximations.

#### Input-output map characteristics revealed by the global classical and inverse metamodels

##### The dominating input-output covariation patterns

**Figure 3:**
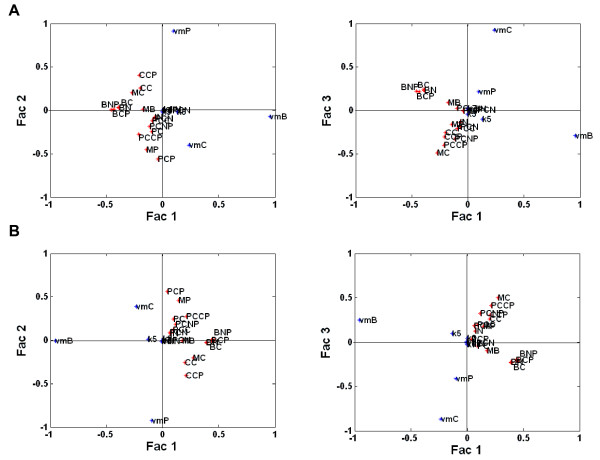
**Maps of the global covariance patterns between the circadian clock state variables and input parameters. **Global NPLSR second mode loadings (Fac 1-Fac 3) for the state variables (red dots) and the parameters (blue dots) from **A**) the *inverse* metamodelling and **B**) the *classical* metamodelling.

In NPLSR, like in other subspace regression methods, the high-dimensional data is projected into a low-dimensional subspace spanned by estimated latent variables that represent the most relevant patterns of input (regressor)-output (response or regressand) covariation (see Additional file 1: Section S1). The couplings between the original variables and the latent variables are called *loadings*. The global relationship patterns between the 9 varied mammalian circadian clock parameters (Table 1) and the 16 state variables (Table 2) were assessed through plots of the first three global second mode NPLSR loadings for the output state variables and the input parameters (Figure 3). Variables placed close to each other in the loading plots are positively correlated, while variables placed opposite each other are negatively correlated in the NPLSR factor space.

**Table 2:** Description of the mammalian circadian clock model state variables

**State variable name**	**Unit**	**Description**
*M*_*P*_	nM	Concentration of Per mRNA
*B*_*N*_	nM	Concentration of non-phosphorylated Bmal1 protein in the nucleus
*M*_*C*_	nM	Concentration of Cry mRNA
*M*_*B*_	nM	Concentration of Bmal1 mRNA
*P*_*C*_	nM	Concentration of non-phosphorylated Per protein in the cytosol
*P*_*CP*_	nM	Concentration of phosphorylated Per protein in the cytosol
*PC*_*C*_	nM	Concentration of non-phosphorylated Per-Cry protein complex in the cytosol
*C*_*C*_	nM	Concentration of non-phosphorylated Cry protein in the cytosol
*C*_*CP*_	nM	Concentration of phosphorylated Cry protein in the cytosol
*PC*_*CP*_	nM	Concentration of phosphorylated Per-Cry protein complex in the cytosol
*PC*_*N*_	nM	Concentration of non-phosphorylated Per-Cry protein complex in the nucleus
*PC*_*NP*_	nM	Concentration of phosphorylated Per-Cry protein complex in the nucleus
*I*_*N*_	nM	Concentration of inactive complex between Per-Cry and Clock-Bmal1 in the nucleus
*B*_*C*_	nM	Concentration of non-phosphorylated Bmal1 protein in the cytosol
*B*_*CP*_	nM	Concentration of phosphorylated Bmal1 protein in the cytosol
*B*_*NP*_	nM	Concentration of phosphorylated Bmal1 protein in the nucleus

The 16 state variables correspond to the 16 ODEs in the mammalian circadian clock model.

Within the parameter space analysed here, the parameters *v*_*mB*_ (maximum rate of Bmal1 mRNA degradation), *v*_*mC*_ (maximum rate of Cry mRNA degradation) and *v*_*mP*_ (maximum rate of Per mRNA degradation) had the highest correlation to the circadian clock state variables along the first three global NPLSR factors, both in the inverse (Figure 3A) and classical (Figure 3B) metamodelling. The same over-all covariation patterns could be seen both in the inverse and the classical global metamodelling, since the orthogonal design in the parameters used in the global metamodelling has no dominant covariance directions, and both the inverse and classical NPLSR metamodels will consequently be dominated by the covariance structures of the state variables (but restricted to the parameter design). The input parameter *v*_*mB*_ were e.g. negatively correlated with the state variables *B*_*C*_, *B*_*N*_, *B*_*NP*_ and *B*_*CP*_ in the NPLSR factor space, which was not surprising since these state variables represent the dynamics of the phosphorylated and non-phosphorylated protein Bmal1 concentrations in the cytosol and nucleus 24. Similarly, *v*_*mP*_ was negatively correlated with the state variables *M*_*P*_ (dynamics of Per mRNA) and *P*_*CP*_ (dynamics of phosphorylated Per protein concentration in the cytosol), while *v*_*mC*_ was negatively correlated with *M*_*C*_ (dynamics of Cry mRNA) and *PC*_*CP*_ (dynamics of phosphorylated Per-Cry complex in the cytosol). These patterns were all in concordance with our intuition of the mammalian circadian clock model.

##### Prediction results from the global inverse metamodelling

**Figure 4:**
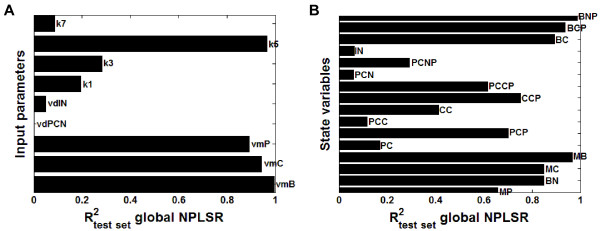
**Test set prediction results from the global NPLSR metamodelling of the mammalian circadian clock model. A**) Results from the test set validation of the *inverse* metamodelling. Correlation coefficient (R^2^)-values from the global NPLSR test set prediction of the parameters from the state variable trajectories are shown, using 19 factors in the global NPLSR model. **B**) Results from the test set validation of the *classical* metamodelling. R^2^-values from the global NPLSR test set prediction of the state variable trajectories from the parameters are shown, using 8 factors in the global NPLSR model.

The test set prediction results from the inverse metamodelling shown in Figure 4A, indicated that the input parameters *v*_*mB*_, *v*_*mC*_*, v*_*mP*_ and *k*_*5*_ (rate constant for entry of the Bmal1 protein into the nucleus) were predicted with reasonably high accuracy (correlation coefficient (R^2^)-values higher than 0.8) from the circadian clock state trajectories, indicating that the circadian clock model was highly sensitive to changes in these input parameters and that the relationship between these parameters and the model output was quite linear. For the input parameters *v*_*dPCN*_, *v*_*dIN*_, *k*_*1*_, *k*_*3*_ and *k*_*7*_, the prediction error was high using global NPLSR metamodelling.

##### Prediction results from the global classical metamodelling

The results from the test set prediction of the state variable trajectories from the input parameters in the classical metamodelling shown in Figure 4B, indicated that the temporal behaviour of the following state variables could be predicted with high accuracy from the input parameters using global NPLSR: *B*_*N*_, *M*_*C*_, *M*_*B*_, *B*_*C*_, *B*_*CP*_ and *B*_*NP*_, while the prediction error was especially high for the state variables *P*_*C*_, *PC*_*C*_, *C*_*C*_, *PC*_*N*_, *PC*_*NP*_ and *I*_*N*_.

**Figure 5:**
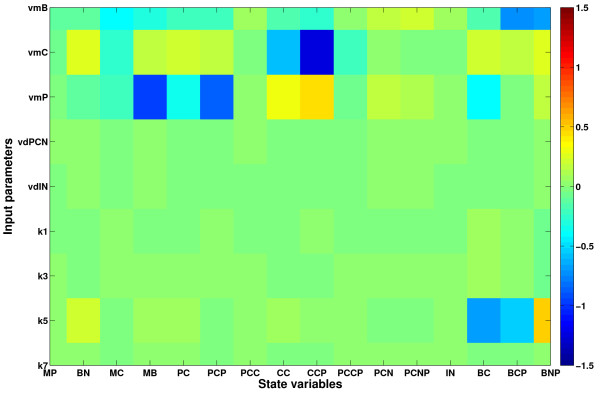
**Mammalian circadian clock model sensitivities estimated from the global classical NPLSR metamodel. **Model sensitivities to variations in the nine varied input parameters calculated as the products between the second mode *X-*factors (*X-*loadings) and the transpose of the second mode ***Y***-factors (***Y***-loadings) from the global classical NPLSR metamodel.

Analogous to the results from the inverse metamodelling described above, the matrix plot of the global NPLSR-estimated sensitivities (estimated as products between the ***X***- and ***Y***- loadings) of the model output state variables to the nine varied parameters in Figure 5 indicated that the circadian clock model was only sensitive to the input parameters *v*_*mB*_, *v*_*mC*_, *v*_*mP*_ and *k*_*5*_. However, the predictive ability obtained with the global metamodelling was not adequate, and important patterns of variation were therefore left un-described. Hence, the parameter-state variable map of the circadian clock model was relatively complex and nonlinear. Since the analysed model was deterministic, we assumed that a higher number of state trajectories would be predicted with high accuracy from the input parameters using a nonlinear metamodel. We therefore hypothesised that hierarchical cluster-based metamodelling would reveal more details of the input-output relationship of the mammalian circadian clock model through regional metamodelling.

#### Separately analysed output space regions in the hierarchical cluster-based metamodelling

To facilitate comparison, it was decided to use the same grouping (clustering) of the observations in both classical and inverse metamodelling. The state variable NPLSR ***X***-factors from the inverse metamodelling were more directly related to the state variable behaviour than the ***Y***-factors representing the state variables in the classical metamodelling due to the asymmetric nature of the NPLSR models (defined primarily based on the ***X***-scores, not the ***Y***-scores). The inverse metamodelling was therefore carried out first, i.e. the clustering of the 8135 calibration set observations was carried out on the inverse metamodelling ***X***-factors obtained from the output state trajectories (***T***_*Output*,*A,Inverse*_, see Figure 2), thereby ensuring that the clusters represented different model behaviours. The same clusters were then also used in the classical N-way HC-PLSR metamodelling. This was chosen due to that clustering on the ***X***-factors or the predicted ***Y***-factors (${\hat{T}}_{\text{YA,NWay}}$) in the classical metamodelling (as would be a more traditional procedure) would both make the clustering more related to the designed parameter combinations instead of the state variable behaviour, since the predicted ***Y***-factors were here predicted as linear combinations of the ***X***-factors that are related to the parameter combinations (see Additional file 1, equation S12c). Predicted ***Y***-factors would have to be used in the clustering instead of the ***Y***-factors directly calculated from the state variable data in the classical metamodelling, since otherwise the classification of new observations (for which state variable data are not available) would not be possible on variables equivalent to those used to cluster the calibration set observations.

**Figure 6:**
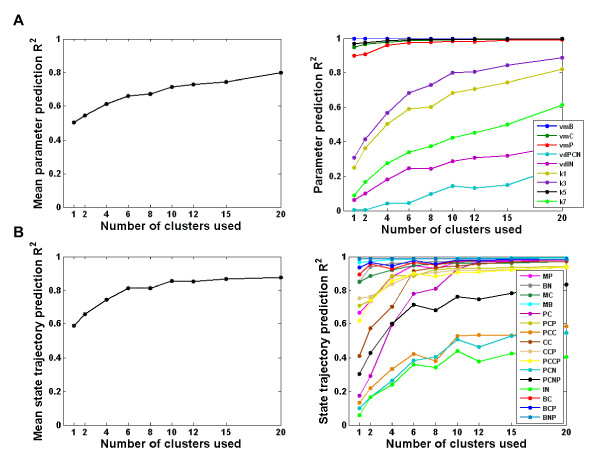
**Optimalisation of the number of clusters in hierarchical metamodelling of the mammalian circadian clock model. A**) Results from *inverse* hierarchical metamodelling using from 1–20 clusters in the N-way HC-PLSR. Left: Mean parameter prediction correlation coefficient (R^2^)-values within the calibration set, over the nine varied circadian clock model input parameters vs. the number of clusters used in the N-way HC-PLSR metamodelling. The calibration set observations were here treated as "new observations" (see Figure 2), and classified in the prediction stage. Using six clusters was considered optimal. Right: Parameter prediction R^2^-values within the calibration set for the nine different circadian clock model input parameters vs. the number of clusters used in the N-way HC-PLSR metamodelling. **B**) Results from *classical* hierarchical metamodelling using from 1–20 clusters in the N-way HC-PLSR. Left: Mean state variable prediction R^2^-values within the calibration set, over the 16 circadian clock model state variables vs. the number of clusters used in the N-way HC-PLSR metamodelling. The calibration set observations were here treated as "new observations", and classified in the prediction stage. Right: State variable prediction R^2^-values within the calibration set for the 16 circadian clock state variables vs. the number of clusters used in the N-way HC-PLSR metamodelling. Using six clusters was considered optimal.

Based on an assessment of the ability to constrain parameters from the state trajectories using from 1 to 20 clusters (Figure 6), using six clusters was considered optimal in order to balance between predictive ability and interpretational complexity. As seen from Figure 6, some of the parameters and state variables could be predicted even more accurately using a higher number of clusters in the N-way HC-PLSR, but that would lead to a more complex model that would be more difficult to interpret in a sensitivity analysis. Keeping the number of clusters as low as possible also helps avoiding overfitting of the data. We therefore assumed that the most important input-output map characteristics could be revealed using six regional NPLSR models in the N-way HC-PLSR.

**Figure 7:**
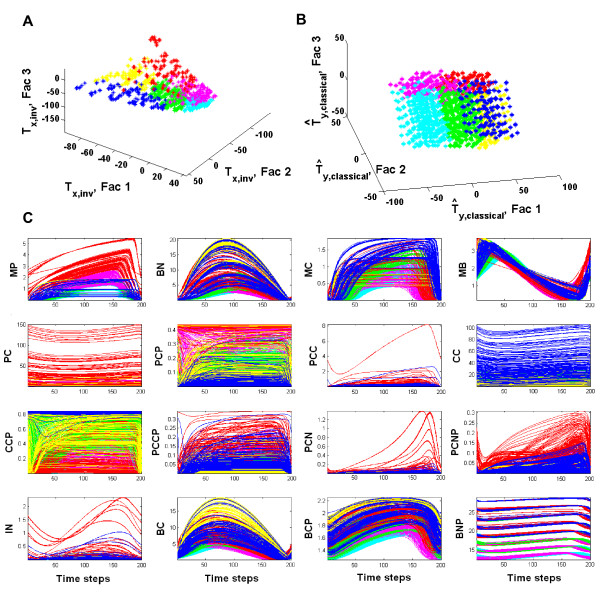
**Clustering results used in the N-way HC-PLSR metamodelling with six clusters. A**) Plot of the $T_{\text{XA,NWay}}$factors (Fac 1-Fac 3) from the global *inverse* metamodelling (=***T***_*Output,A,Inverse*_). The observations are coloured according to cluster memberships. Cluster1=blue, cluster2=red, cluster3=yellow, cluster4=green, cluster5=magenta, cluster6=cyan. ***X*** is the 3-way state variable trajectory array, while *Y* is the parameters. The clustering was done on the $T_{\text{XA,NWay}}$factors explaining a significant amount of the variation in the state variable space, that is the 19 first factors. **B**) Plot of the predicted ***Y****-*scores ${\hat{T}}_{\text{YA,NWay}}$ (see Additional file 1, eq. S12c) from the global *classical* metamodelling, colour coded according to the cluster memberships of the observations found using ***T***_*Output,A,Inverse*_. The classification of the test set observations to be predicted in the classical metamodelling was based on ${\hat{T}}_{\text{Output,A,Inverse}}$, predicted from ${\hat{T}}_{\text{YA,NWay}}$ using second order polynomial OLS regression. This OLS prediction model was calibrated in the calibration step of the classical metamodelling, based on the $T_{\text{XA,NWay}}$factors from the inverse metamodelling (=$T_{\text{Output,A,Inverse}}$, plotted in panel A) and calibration set ${\hat{T}}_{\text{YA,NWay}}$ (plotted here). **C**) Circadian clock state trajectories for the observations belonging to each cluster, coloured according to cluster memberships from the inverse N-way HC-PLSR. Cluster1 = blue, cluster2 = red, cluster3 = yellow, cluster4 = green, cluster5 = magenta, cluster6 = cyan. All state variables are given in nM units.

The clustering of the calibration set observations used in the final N-way HC-PLSR metamodelling is illustrated in Figure 7 both in the NPLSR factor spaces from the inverse (Figure 7A) and classical (Figure 7B) metamodelling and in the original state variable trajectory space (Figure 7C). Figure 7B illustrates that the NPLSR ***Y***-factors from the classical metamodelling were (as expected) highly related to the designed parameter combinations, and hence did not give as good representation of the state variable behaviour as the ***X***-factors from the inverse metamodelling. As described above, the clustering was therefore based on the latter both in the classical and the inverse metamodelling. Figure 7C confirmed that the six clusters represented different types of dynamic behaviour for the mammalian circadian clock model. For example, Cluster 1 was characterised by e.g. an especially large spread in the values of the state variable *C*_*C*_, while Cluster 2 was characterised by high values of several of the circadian clock state variables (especially *M*_*P*_, *P*_*C*_, *P*_*CP*_, *PC*_*C*_, *PC*_*N*_, *PC*_*NP*_ and *I*_*N*_). The parameter ranges for the clusters are given in Table 3, and showed that the clustering of the observations had a close relation to the values of the parameters *v*_*mB*_, *v*_*mC*_ and *v*_*mP*_, which also spanned the first three global NPLSR factors both in the inverse and classical metamodelling.

**Table 3:** Parameter ranges and mean values for the observations in the six N-way HC-PLSR clusters

**Cluster**	***v***_***mB***_**(nMh**^**-1**^**)**	***v***_***mC***_**(nMh**^**-1**^**)**	***v***_***mP***_**(nMh**^**-1**^**)**	***v***_***dPCN***_**(nMh**^**-1**^**)**	***v***_***dIN***_**(nMh**^**-1**^**)**	***k***_***1***_**(h**^**-1**^**)**	***k***_***3***_**(nM**^**-1**^ **h**^**-1**^**)**	***k***_***5***_**(h**^**-1**^**)**	***k***_***7***_**(nM**^**-1**^ **h**^**-1**^**)**
1	0.02-0.23 (0.11)	0.95-1.12 (0.99)	1.14-2.09 (1.65)	0.99-1.14 (1.06)	0.08-1.52 (0.81)	0.08-1.52 (0.78)	0.08-1.52 (0.82)	0.27-0.41 (0.34)	0.05-0.95 (0.49)
2	0.02-0.28 (0.11)	0.95-1.54 (1.26)	0.98-1.14 (1.02)	0.99-1.14 (1.06)	0.08-1.52 (0.77)	0.08-1.52 (0.78)	0.08-1.52 (0.83)	0.27-0.41 (0.34)	0.05-0.95 (0.49)
3	0.02-0.07 (0.04)	1.03-1.54 (1.32)	1.14-2.09 (1.65)	0.99-1.14 (1.07)	0.08-1.52 (0.80)	0.08-1.52 (0.81)	0.08-1.52 (0.77)	0.27-0.41 (0.32)	0.05-0.95 (0.51)
4	0.07-0.33 (0.18)	0.95-1.54 (1.25)	1.14-2.09 (1.70)	0.99-1.14 (1.07)	0.08-1.52 (0.80)	0.08-1.52 (0.79)	0.08-1.52 (0.81)	0.27-0.41 (0.34)	0.05-0.95 (0.50)
5	0.12-0.38 (0.27)	0.95-1.54 (1.29)	0.98-1.30 (1.09)	0.99-1.14 (1.06)	0.08-1.52 (0.80)	0.08-1.52 (0.82)	0.08-1.52 (0.79)	0.27-0.41 (0.34)	0.05-0.95 (0.50)
6	0.23-0.38 (0.33)	0.95-1.54 (1.29)	1.14-2.09 (1.69)	0.99-1.14 (1.07)	0.08-1.52 (0.80)	0.08-1.52 (0.80)	0.08-1.52 (0.79)	0.27-0.41 (0.35)	0.05-0.95 (0.51)

The mean values are given in parenthesis, while the ranges give the minimum and maximum parameter values observed in each cluster.

#### Additional input-output map characteristics revealed by the regional classical and inverse metamodelling

##### Prediction results from the hierarchical inverse metamodelling

**Figure 8:**
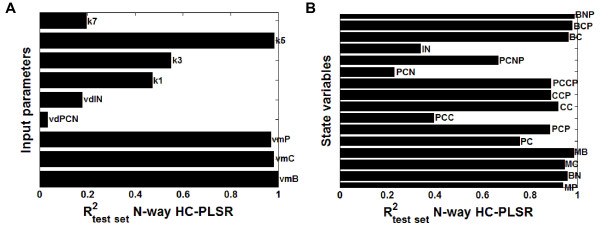
**Prediction results from the hierarchical inverse and classical metamodelling. A**) R^2^-values from the hierarchical NPLSR test set prediction of the parameters from the state variable time series using six regional regression models, using 18, 19, 19, 18, 19 and 17 NPLSR factors, respectively. The clustering was done on the global $T_{\text{XA,NWay}}$ factors, using 19 factors. **B**) R^2^-values from the hierarchical NPLSR test set prediction of the state variable trajectories from the parameters using six regional regression models, all using 9 NPLSR factors. The same clusters as in the inverse metamodelling were used.

The test set prediction results from the hierarchical inverse metamodelling shown in Figure 8A indicated that the two input parameters *k*_*1*_ (rate constant for entry of the Per-Cry complex into the nucleus) and *k*_*3*_ (rate constant for the formation of the Per-Cry complex) were predicted with considerably higher accuracy in the hierarchical metamodelling compared to the global metamodelling. Figure 6 indicated that increasing the number of clusters in the N-way HC-PLSR had a large effect on the prediction accuracy for these two parameters; R^2^-values higher than 0.8 could be achieved using 20 clusters. However, the increase in prediction accuracy obtained also using only six clusters indicated that the circadian clock model was sensitive to these two parameters, in contrast to what the global metamodelling indicated. Hence, the hierarchical metamodelling could provide additional insights into the input-output map of the analysed model.

The three parameters *v*_*dPCN*_ (maximum rate of degradation of nuclear phosphorylated Per-Cry complex), *v*_*dIN*_ (maximum rate of degradation of nuclear Per-Cry-Clock-Bmal1 complex) and *k*_*7*_ (rate constant for the formation of the inactive Per-Cry-Clock-Bmal1 complex) were predicted with low accuracy also in the inverse hierarchical metamodelling. This indicated that either the circadian clock model was relatively insensitive to variations in these input parameters, or our metamodelling was not able to describe the complex relationships between these parameters and the model outputs. This has been assessed in more detail below.

##### Prediction results from the hierarchical classical metamodelling

Several of the circadian clock state variable trajectories could be predicted with considerably higher accuracy in classical metamodelling using N-way HC-PLSR compared to the global NPLSR (Figure 8B). Only the state variables *PC*_*C*_ (concentration of the Per-Cry protein complex in the cytosol), *PC*_*N*_ (concentration of the Per-Cry protein complex in the nucleus) and *I*_*N*_ (concentration of the inactive complex between Per-Cry and Clock-Bmal1 in the nucleus) were predicted with low accuracy (R^2^-values below 0.4), indicating that the metamodelling with N-way HC-PLSR was able to capture the main features of the input-output mappings for most of the 16 circadian clock state variables.

Figure 7 indicated that the data for the three above mentioned state variables contained extreme outliers (especially in Cluster 1 and 2), which may have caused the low prediction accuracy for these state variables. Both for the calibration set and the test set, being an outlier seemed to be closely associated to having the lowest level of the parameter *v*_*mP*_, but this was not itself sufficient to generate extreme values of these three state variables.

##### Detailed interpretation of the revealed model sensitivity patterns

**Figure 9:**
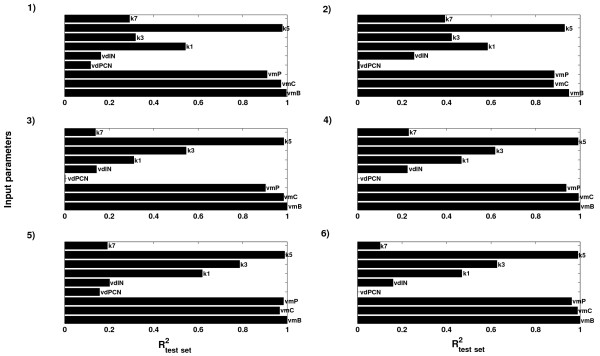
**Prediction results from hierarchical *inverse *metamodelling within each regional regression model in the N-way HC-PLSR. **The R^2^-values from test set prediction of the parameters from the state variables are shown for regional model 1–6, corresponding to the clusters used in the N-way HC-PLSR. The regional models use 18, 19, 19, 18, 19 and 17 NPLSR factors, respectively. The clustering was done on the global $T_{\text{XA;NWay}}$factors, using 19 factors.

**Figure 10:**
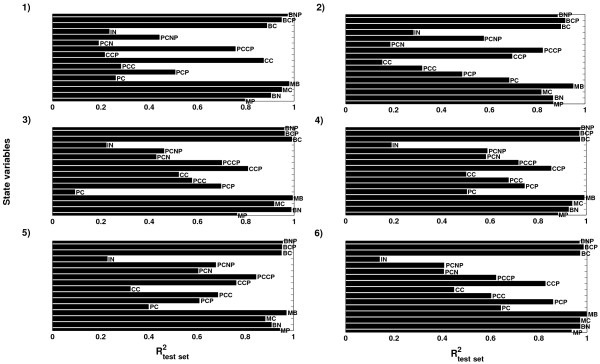
**Prediction results from hierarchical *classical *metamodelling within each regional regression model in the N-way HC-PLSR. **The R^2^-values from test set prediction of the state variable trajectories from the parameters are shown for regional model 1–6, corresponding to the clusters used in the N-way HC-PLSR. All regional models use 9 NPLSR factors. The same clusters as in the inverse metamodelling were used.

**Figure 11:**
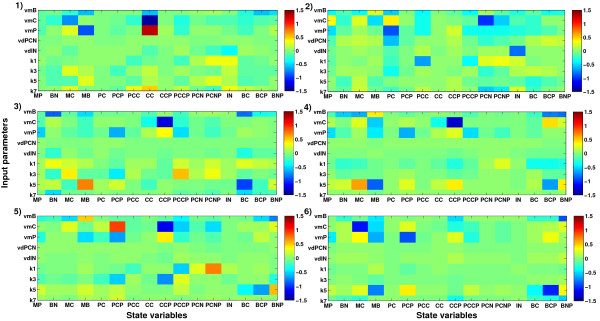
**Mammalian circadian clock model sensitivities estimated from each of the regional *classical *NPLSR metamodels. **Model sensitivities to variations in the nine varied input parameters calculated as the products between the second mode *X-*factors and the transpose of the second mode ***Y***-factors from regional NPLSR model 1–6 in the N-way HC-PLSR metamodelling.

The parameter- and state variable prediction results within each of the regional NPLSR metamodels, shown in Figure 9 and Figure 10, indicated clear regional differences in the state variable space with regard to the prediction accuracy for the different parameters and state trajectories. This may be used to identify the parameters that are especially important for certain types of model behaviour. We recently showed that regional differences in model sensitivity to the input parameters also represent complex interaction patterns between the parameters 9. In order to illustrate the usefulness of the methodology in providing biological insight, we give below some examples of detailed interpretations of the sensitivity patterns illustrated in Figures 9, 10 and 11.

As shown in Figure 10, the temporal behaviours of the state variables *PC*_*C*_ and *PC*_*N*_ were predicted with higher accuracy by all regional metamodels except regional model 1 and 2 (corresponding to clusters containing outliers for these state variables), compared to the global NPLSR. However, the state variable *I*_*N*_ was predicted with very low accuracy in all the regional metamodels, and the parameters *v*_*dPCN*_, *v*_*dIN*_ and *k*_*7*_ could not be well predicted in any of the regional inverse metamodels (Figure 9). Two of the parameters that were predicted with low accuracy, *v*_*dIN*_ and *k*_*7*_, appeared in the differential equation corresponding to the state variable *I*_*N*_. Hence, the low prediction accuracy was probably due to an insufficiently described mapping between *I*_*N*_ and these parameters in the N-way HC-PLSR.

In order to reveal the sensitivity patterns for the state variable *I*_*N*_, a separate sensitivity analysis of the relationship between *I*_*N*_ and the circadian clock input parameters was carried out using 2-way second order polynomial HC-PLSR analogous to the analysis presented in 8, but with the parameter ranges given in Table 1. This showed that in order to be well-predicted, the state variable *I*_*N*_ had to be logarithmised prior to the analysis. This might explain why this state variable trajectory could not be well described together with the other state variables in the N-way HC-PLSR. The results are given in Additional file 1: Section S3.1, and indicated that the parameters having the largest effects on the *I*_*N*_ state trajectory were *v*_*mB*_, *v*_*mC*_, *v*_*mP*_, *v*_*dIN*_, *k*_*1*_ and *k*_*7*_. Several interactions between these input parameters were also identified.

The parameter *k*_*7*_ (rate constant for the formation of the inactive Per-Cry-Clock-Bmal1 complex) was also involved in the differential equations representing the dynamics of the concentration of non-phosphorylated Bmal1 protein in the nucleus (state variable *B*_*N*_) 24 and the concentration of the non-phosphorylated Per-Cry protein complex in the nucleus (state variable *PC*_*N*_), in addition to *I*_*N*_. *PC*_*N*_ was not well described by the classical metamodelling, but *B*_*N*_ was predicted with high accuracy from the parameters (Figure 8B and Figure 10). Hence, the low prediction accuracy for *k*_*7*_ indicated that the state variable *B*_*N*_ was relatively insensitive to the parameter *k*_*7*_ according to our analysis (within the analysed parameter range), even though the differential equation for this state variable involved *k*_*7*_. This was confirmed by the plot of the model sensitivities estimated from the regional metamodels shown in Figure 11 (only in regional model 3 a sensitivity was identified, but this was also low), as well as the separate sensitivity analysis for the state variable *B*_*N*_ described in Additional file 1: Section S3.2 (carried out in the same way as for *I*_*N*_). This illustrates how a combination of a classical and inverse metamodelling can provide more confident conclusions about model behaviour and sensitivity patterns.

The third parameter that could not be constrained from the state variable data was *v*_*dPCN*_ (maximum rate of degradation of nuclear phosphorylated Per-Cry complex), which was only involved in the differential equation describing the dynamics of the concentration of the phosphorylated Per-Cry complex in the nucleus (state variable *PC*_*NP*_). This state variable was predicted with an R^2^-value of approximately 0.7 in the classical metamodelling, which is not particularly low. Thus our results indicated a low sensitivity of *PC*_*NP*_ to the rate of degradation of the corresponding protein. This result was confirmed by the results shown in Figure 11 and the separate sensitivity analysis for the state variable *PC*_*NP*_ described in Additional file 1: Section S3.3. This was not straight-forward to explain, and calls for a more comprehensive analysis of the relationship between the state variable *PC*_*NP*_ and the input parameter *v*_*dPCN*_. Possible explanations might be that our analysis did not cover the relevant range for this parameter, causing the model sensitivity to this parameter not to be detected, or that its input-output relationship is very complex.

As seen from Figure 11, the parameter *v*_*dPCN*_ seemed to have negative impact on the state variable *P*_*C*_ (concentration of non-phosphorylated Per protein in the cytosol) in regional NPLSR model 2, even though neither this parameter nor the state variable *PC*_*NP*_ (concentration of the phosphorylated Per-Cry complex in the nucleus, for which this parameter was involved in the differential equation), appeared in the differential equation representing *P*_*C*_. Both *P*_*C*_ and *PC*_*NP*_ were related to the Per protein, though. In this region of the state variable space, the effect of *v*_*dPCN*_ on *P*_*C*_ was either more pronounced, or the relationship between these variables was less complex and therefore more visible in the analysis. As seen from Figure 7C, Cluster 2 was characterised by high values of several of the circadian clock state variables (especially *M*_*P*_, *P*_*C*_, *P*_*CP*_, *PC*_*C*_, *PC*_*N*_, *PC*_*NP*_ and *I*_*N*_).

The input parameter *k*_*3*_ (rate constant for the formation of the Per-Cry complex) had a large negative effect on *C*_*CP*_ that was visible only in Cluster 5 (Figure 11). This could be explained by the fact that *k*_*3*_ was involved in the equation for *C*_*C*_, which was part of the differential equation for *C*_*CP*_. Furthermore, *v*_*dIN*_ (maximum rate of degradation of nuclear Per-Cry-Clock-Bmal1 complex) seemed to have a slight negative effect on *PC*_*NP*_ in Cluster 1. This result was not easily deducible from the equation structure of the circadian clock model, and could not be detected in the global metamodelling. Cluster 1 was characterised by e.g. an especially large spread in the values of the state variable *C*_*C*_.

The parameter *k*_*7*_ (rate constant for the formation of the inactive Per-Cry-Clock-Bmal1 complex) seemed to have a positive effect on the state variable *C*_*C*_ (non-phosphorylated Cry protein in the cytosol) in regional metamodel 1. However, since the inactive Per-Cry-Clock-Bmal1 complex represses the Per and Cry genes in the nucleus, a positive effect of *k*_*7*_ on *C*_*C*_ seemed unlikely. An additional sensitivity analysis was therefore carried out by adding eight simulations to the data set, keeping all parameters except *k*_*7*_ constant at the mean values found for Cluster 1. The results are shown in Additional file 1: Section S3.4, and indicated that increasing *k*_*7*_ resulted in a very small decrease in *C*_*C*_ and *C*_*CP*_, had a clear positive effect on *I*_*N*_ (as expected from the differential equation for *I*_*N*_), and a negative effect on *PC*_*N*_ and *PC*_*NP*_. In order to try to explain the positive effect of *k*_*7*_ on *C*_*C*_ seen in Cluster 1, a separate 2-way PLSR-based sensitivity analysis was therefore also carried out for the state variable *C*_*C*_ in Cluster 1 (see Additional file 1: Section S3.4). The effects of *v*_*mB*_, *v*_*mC*_, *v*_*mP*_ and *k*_*3*_ on *C*_*C*_ indicated for Cluster 1 were also manifested in the 2-way PLSR analysis, but a positive main effect of *k*_*7*_ was not confirmed. However, several interaction terms involving *k*_*7*_ seemed to have effects on *C*_*C*_, such as the interaction between *v*_*mP*_ and *k*_*7*_ (which had a positive effect). Since cross-terms between the input parameters were not included in the N-way PLSR analysis, confounding of these interaction effects with the main effect of *k*_*7*_ may explain the positive sensitivity to *k*_*7*_ indicated by the N-way PLSR. The indication of a positive effect of *k*_*7*_ on *C*_*C*_ could also have been caused by other sources of uncertainties in the NPLSR analysis.

Analogous to the increased prediction accuracy obtained for the two parameters *k*_*1*_ and *k*_*3*_, model sensitivity to these parameters could be revealed in several local regions of the state variable space (Figure 11), even though this was not evident from the global metamodelling (Figure 5). According to Figure 11, the model seemed to be insensitive to these parameters in the region represented by Cluster 6. However, the inverse metamodelling indicated that also in Cluster 6 these parameters could be predicted with a much higher accuracy than by the global NPLSR metamodel, indicating model sensitivity to these parameters also in this region of the state variable space. This illustrates the importance of carrying out both classical and inverse metamodelling to gain a more detailed insight into the sensitivity patterns of a complex model.

## Discussion

The main traditional approach to analysis of input-output relationships has been to use aggregated outputs derived from the state trajectories, representing the dynamics of the state variables. For instance, in their original publication of the mammalian circadian clock model 24, the authors employed a sensitivity analysis of only one aggregated output – the circadian clock period– a very important trait, but too aggregated to give sufficient overview of the entire model behaviour. Multivariate metamodelling has, at least in principle, the capacity to reveal the relationships between all input parameters and all model outputs simultaneously. This has here been illustrated for the nine input parameters assumed to be most interesting for the mammalian circadian clock and the 16 state variables of the model, where the generated N-way metamodels allowed flexible quantitative input-output regressions yielding informative graphical insight into the main underlying input-output map characteristics. In our example N=3, but the analysis can be extended to more than three modes.

Our analysis confirmed the main conclusions from the original classical sensitivity analysis of the circadian clock period carried out by Leloup and Goldbeter 24, namely that the mammalian circadian clock model was highly sensitive to parameters related to synthesis and degradation of the protein Bmal1 and its mRNA. However, our analysis improved the overview of the input-output relationships on which the circadian clock period is based. The main patterns found in our previous analysis of the same model, using conventional (2-way) PLSR 7, were also confirmed in the global NPLSR metamodelling. However, the present cluster-based N-way analysis revealed additional aspects of the input-output relationships, for example the negative effect of increasing *v*_*dPCN*_ on the state variable *P*_*C*_ in the part of the model output space defined by Cluster 2. Hence, the N-way HC-PLSR-based metamodelling worked as intended in this illustration example. In the example used here, the focus was on oscillating state variables. Other types of behaviour of nonlinear dynamic systems such as multiple steady states could potentially lead to additional nonlinearities in the input-output mapping, probably increasing the gain of using a cluster-based approach compared to a global analysis.

An alternative to using NPLSR would be to unfold the state variable trajectory array by concatenating all the trajectory data into one 2-way matrix and use 2-way HC-PLSR to analyse the data. However, the information about related trajectories for different state variables would then be left unused, leading e.g. to loss of the opportunity to visualise covariance structures. In order to evaluate the gain of keeping the 3-way structure in the data, the same analysis was carried out using 2-way HC-PLSR on unfolded state trajectory data as well as on aggregated outputs calculated from the state trajectories. The clustering results from these analyses (shown in Additional file 1: Section S4) indicated that the increased resolution achieved using N-way HC-PLSR could not be achieved when the state trajectory array was unfolded into two dimensions or transformed into aggregated outputs, due to a less reasonable clustering of the observations. Hence, using the NPLSR factors seems to enable identification of more relevant clusters in which to carry out regional metamodelling. The global parameter prediction accuracies obtained were comparable to those obtained with the global inverse N-way PLSR. However, in the hierarchical metamodelling, neither the unfolded state trajectories nor the aggregated outputs could predict the circadian clock parameters with as high accuracy (on average) with 2-way HC-PLSR as with the N-way HC-PLSR.

In contrast to the results obtained using N-way HC-PLSR, our previously published metamodelling of each of the circadian clock state variables separately 8 showed that all circadian clock state variables could be predicted with high accuracy from the parameters (within the parameter space analysed in that publication, which was slightly different in the present analysis). However, there is a clear gain of using a common metamodel for all state variables in terms of obtaining overview of the input-output relationships as well as covariance patterns between the state variables. Nevertheless, as demonstrated here, a separate analysis of the input-output relationships for insufficiently described state variables should accompany this type of analysis in order to gain a more complete insight into the input-output relationships. This was illustrated in our application example for e.g. the state variable *I*_*N*_, which had to be logarithmised and analysed separately in order for its relationships to the input parameters to be adequately described.

In NPLSR, relations between model outputs and input parameters are easily interpretable through plots of the loadings, in contrast to results produced e.g. by genetic algorithms which are often more difficult to interpret (although the latter can also handle multiple outputs). Moreover, due to the decomposition of the data into estimated latent variables, NPLSR can provide efficient dimension reduction possibilities in high-dimensional systems. However, since the NPLSR models presented here used a high number of factors to explain the input-output covariance, the dimension reduction possibilities of NPLSR may not have been fully utilised. This was caused by the differences in the time-to-peak for the different state variables, which the NPLSR uses many factors to describe. Hence, a more careful pre-processing of the data would probably result in NPLSR models using fewer factors, perhaps through shift correction as described by Westad and Martens 29. Work is in progress on testing whether this allows the NPLSR models to use fewer factors while still keeping the same predictive ability. However, even when using relatively many factors, the NPLSR models still enable great dimension reduction possibilities.

In regional regression modelling, there is a risk that the variance in some input- or output variables is highly reduced in the regional models compared to the entire data set. Hence, the robustness of the predictions may decrease and the regression coefficients as well as the R^2^-values may be misleading for these variables. However, as shown in Table 4, the criterion used for defining local clusters (based on fuzzy clustering searching for spherical clusters) ensured that variances in the nine input parameters did not differ much between the clusters, and were about the same in the calibration set and the test set; the only parameters for which the variance decreased in the clusters compared to in the original datasets were *v*_*mB*_, *v*_*mC*_ and *v*_*mP*_. This was not surprising, since these three parameters had the largest impacts on the first three NPLSR factors of the global NPLSR models, and hence the clustering using the NPLSR factors was mostly based on these three parameters. However, since these three parameters were also predicted with high R^2^-values in the global inverse metamodel, high R^2^-values were not artefacts of low cluster variance in this study. This is primarily a problem occurring when using small test sets, and here the test set was of approximately the same size as the calibration set (more than 8000 simulations in each).

**Table 4:** Parameter value variances in the calibration and test set and the six N-way HC-PLSR clusters

**Data set**	***v***_***mB***_	***v***_***mC***_	***v***_***mP***_	***v***_***dPCN***_	***v***_***dIN***_	***k***_***1***_	***k***_***3***_	***k***_***5***_	***k***_***7***_
Calibr. set	0.0138	0.0373	0.1308	0.0024	0.2225	0.2225	0.2220	0.0021	0.0869
Cluster 1	0.0041	0.0030	0.0936	0.0024	0.2340	0.1916	0.2166	0.0016	0.0819
Cluster 2	0.0044	0.0348	0.0046	0.0024	0.2391	0.2338	0.2133	0.0019	0.0890
Cluster 3	0.0006	0.0225	0.0854	0.0024	0.2065	0.2252	0.2196	0.0017	0.0869
Cluster 4	0.0043	0.0320	0.0714	0.0024	0.2268	0.2328	0.2330	0.0025	0.0895
Cluster 5	0.0061	0.0347	0.0095	0.0024	0.2165	0.2211	0.2232	0.0022	0.0848
Cluster 6	0.0025	0.0326	0.0787	0.0024	0.2186	0.2217	0.2164	0.0019	0.0871
Test set	0.0139	0.0373	0.1289	0.0024	0.2220	0.2272	0.2203	0.0021	0.0871

Since the selection of data subsets in N-way HC-PLSR is based on fuzzy clustering, no prior knowledge about the structure of the data is needed. Hence, this method automatically detects regions of different model behaviour. The number of clusters to use in the hierarchical metamodelling was here specified in advance, based on exploration of the predictive ability of metamodels of varying complexity. However, using instead an optimisation algorithm to find the optimal number of clusters would make semi-automatic exploration of input-output relationships of computational models possible.

## Conclusions

The N-way HC-PLSR method presented here provides the opportunity to improve both prediction accuracy and analytical insight by identification of regional subsets of the data within which the relationships between input parameters and model outputs are more transparent than in a global regression analysis. This was exemplified by the model sensitivity to the two parameters *k*_*1*_ and *k*_*3*_ that was detected in the regional analysis but not in the global metamodelling.

Our results also indicate that analysing all state trajectories simultaneously using N-way methodology is more effective for identification of different behavioural domains for a system and regions where input-output mappings can be predicted with higher accuracy, than unfolding the state trajectory array into two dimensions or transforming state trajectories into aggregated outputs prior to the analysis. This is due to a more reasonable clustering of the observations. Moreover, application of the method for metamodelling in both the classical and the inverse direction represents a more comprehensive approach to the analysis of complex relationships between the model inputs and the temporal behaviour of the outputs, and allows more confident conclusions. Our results showed that the mammalian circadian clock model was highly sensitive to parameters related to the protein Bmal1, as previously found by Leloup and Golbeter 24, but in addition our approach revealed also more complex sensitivity patterns of the model.

Based on these results, we believe that the presented N-way HC-PLSR method will be instrumental for effective construction and validation of complex models. Due to its efficient handling of N-way data structures, demonstrated here in the analysis of the temporal model behaviour, we hypothesise that N-way HC-PLSR will be an especially useful tool for multivariate metamodelling of spatiotemporal models, a large future application area.

## Methods

### Generation of the *in silico* data set

A model of the mammalian circadian clock developed by Leloup and Goldbeter 24 was used to estimate the circadian oscillations of cellular activity in conditions of continuous darkness. The model consists of 16 coupled differential equations with state variables describing the dynamics of three key genes (Bmal1, Per and Cry), including their mRNA level, nonphosphorylated and phosporylated proteins as well as protein complexes. The model contains intertwined positive and negative feedback loops driving the circadian oscillations. A curated CellML implementation 30,31,32 of the model was downloaded from http://models.cellml.org. The integration was carried out in SUNDIALS 2.3 33 using a wrapper for PySundials (http://pysundials.sourceforge.net) in the same way as in 8.

The parameter combinations in the calibration set were generated using an Optimised Multi-level Binary Replacement (OMBR) Design 26 of 9 variables with 8 equally spaced levels each (Table 1). This resulted in 8192 simulations with the circadian clock model. The ranges of each parameter are given in Table 1, and were found by an initial range-finding experiment published in 7. A full factorial design of 9 variables Â´a 8 levels would require 8^9^>134 million runs. Hence, the OMBR design was chosen, in order to explore the effects of as many parameters and parameter values as possible. In the OMBR design method, the values of a original parameters are replaced by multi-bit binary representations, and the binary factor bits are then combined in a fractional factorial design according to a chosen confounding pattern. Thereby drastically reduced experimental designs are obtained, yet maintaining reasonable coverage of the parameter space. The OMBR design has been compared to central composite designs and semi-random designs, and has been shown to give good predictive ability 7.

For each parameter combination the resulting differential equation model was solved from the original initial conditions (see 24) until convergence to a stable limit cycle. The test for convergence was done as follows: First the system was solved with rootfinding for variable *M*_*B*_ to extract two complete cycles. Convergence of the cycle period was tested by requiring that the period difference relative to the mean of the periods for the two cycles should be less than 0.001. Convergence to synchronous oscillations was tested by (i) interpolating all 16 state variables at 200 equally spaced time points for each cycle, (ii) linearly transforming each state variable such that the minimum and maximum values of each cycle was 0 and 1, respectively, and (iii) requiring that the sum of absolute difference between the two cycles across all the 3200 interpolated time points should be less than 0.0001.

The data set resulting from the simulations of the mammalian circadian clock consisted of sampled values for one period (here 200 timesteps) of 16 state variables (corresponding to the 16 differential equations in the model), for the set of 8192 combinations of values for the nine varied input parameters. This gave a 3-way array of 8192x16x200 data points. A description of the mammalian circadian clock model state variables is given in Table 2. Due to the wide parameter ranges used, 57 (0.7%) of the 8192 simulations did not result in a stable limit cycle. These simulations were omitted in the following analysis. The parameters and state variables were mean-centred and standardised globally in the calibration set prior to the metamodelling.

A separate test set based on 8192 parameter combinations found by random Monte Carlo sampling 27,28 within the same parameter levels as used in the calibration set was used. This resulted in 8125 converging test set simulations. In the test set, the variables were pre-processed in the same way as for the calibration set, using the global calibration set means and standard deviations.

### N-way HC-PLSR

Our previously published method for nonlinear metamodelling, HC-PLSR 8, has here been extended to enable use of N-way data by using NPLSR 19,34, giving N-way HC-PLSR. HC-PLSR 8 includes regional analysis using subsets of the original data set generated by fuzzy *C*-means (FCM) clustering 35,36,37,38 (see Additional file 1: Section S1).

In N-way HC-PLSR, a global NPLSR model comprising all observations is first generated, and FCM clustering (using Euclidian distance) on a chosen number of first mode (see Figure 1) factors (scores) of the global NPLSR ***X***-factors (or alternatively, ***Y***-factors) is used to separate the observations into groups within which regional NPLSR models are made. The FCM fuzzifier parameter was here chosen equal to 2. To prevent possibly unstable regression models due to a small number of calibration observations, we post-processed the clustering with the requirement that each cluster should contain at least ten observations. Smaller clusters (and their associated observations) were regarded as outliers, and not included in the subsequent regional regression analysis (but were still included in the global regression analysis).

The optimal number of factors to use in the global and regional NPLSR models, respectively, was here chosen according to the minimum cross-validated mean squared error (MSE) of prediction of the response array ***Y***, with the extra requirement that each included component accounts for at least 1% of the total cross-validated ***Y***-variance, in the same way as in 8. Here, 10-fold cross-validation where the ten segments were randomly chosen was used both in the global and the regional NPLS regressions. These ten segments were successively kept out of the NPLSR calibration, and predicted using an NPLSR model based on the remaining observations. NPLSR is described in Additional file 1: Section S1, and the MATLAB® N-way Toolbox v.3.11 34 was used here.

In our N-way HC-PLSR implementation, Linear Discriminant Analysis (LDA) 39, Quadratic Discriminant Analysis (QDA) 40 (the MATLAB® function "classify" from the Statistics Toolbox™ v7.6) or Naive Bayes classification (the MATLAB® function "NaiveBayes" from the Statistics Toolbox™ v7.6) can be used for classification of new observations to be predicted (based on predicted NPLSR factors for new observations). The implementation contains two options for prediction: 1) Prediction using the local regression model calibrated in the most probable cluster, and 2) Prediction using a weighted sum of the local regression models, using the estimated cluster membership values as weights. The N-way HC-PLSR was carried out in MATLAB® 41 Version 7.13 (R2011b), using in-house code which can be obtained from the authors upon request.

### Classical and inverse metamodelling of the mammalian circadian clock model

The 3-way array of state variable data (observations x outputs x time points) was first used as regressor in a test set validated N-way HC-PLSR using the parameter combinations as response-variables (inverse metamodelling). To complement this analysis, analogous classical metamodelling with N-way HC-PLSR was carried out, where the parameter combinations were used as regressor variables to predict the 3-way state trajectory array. The calibration- and test sets calculated with the mammalian circadian clock model described above were used. The methodology is illustrated in Figure 2. The most probable local regression model was chosen for prediction, since this gave higher prediction accuracy than using a weighted sum of the local models. No clusters containing less than ten observations were identified, so all calibration set observations were included in the regional regression analysis.

In the inverse metamodelling, the clustering (and classification in the prediction stage) of the observations was based on the global predicted first mode ***X****-*factors, the ***X****-*scores ($T_{\text{Qutput,A,Inverse}}=T_{\text{XA,NWay}}$ in Additional file 1: Section S1), representing the state variable trajectory data. All factors found to explain a significant amount of the cross-validated calibration set *Y*-variation were included. The number of clusters to use was chosen by evaluating the ability to constrain (i.e. correctly predict) the input parameters from the model output in the inverse metamodelling, using from 1 to 20 clusters within the calibration set. The calibration set observations were treated as "new observations" (see Figure 2) here, that is, the same procedure as for the test set observations was used in the prediction stage. Using cross-validation to find the optimal number of clusters, as was done to find the optimal number of factors for the NPLSR models, would be too time consuming given the size of the datasets used here. The mean correlation coefficient (R^2^)-values for the prediction of the parameters were used as selection criterion, and using 6 clusters was considered optimal in order to balance metamodel complexity against predictive ability.

The same clusters were also used for the classical metamodelling, since the ***X***-factors in the inverse metamodelling are more directly related to the model output state variables than the ***Y***-factors from the classical metamodelling (a PLSR model is asymmetric). However, for the classification in the prediction stage of the classical metamodelling to be relevant, ${\hat{T}}_{\text{Qutput,A,Inverse}}$ was predicted from the first mode predicted ***Y***-factors, the ***Y***-scores ${\hat{T}}_{\text{YA,NWay}}$ (calculated using equation S12c in Additional file 1), since in this case the ***Y***-factors represent the state variable trajectories. This was done using second order polynomial Ordinary Least Squares (OLS) regression (including square terms and cross-terms), calibrated using calibration set ${\hat{T}}_{\text{YA,NWay}}$ from the classical metamodelling ($=T_{\text{Input,A,Classical}}$* *C*_*A*_ (see Additional file 1, eq. S12c)) as regressors and calibration set ***T***_*XA,NWay*_ from the inverse metamodelling ($=T_{\text{Qutput,A,Inverse}}$) as response variables. Hence, ${\hat{T}}_{\text{Qutput,A,Inverse}}=\hat{F}\left({\hat{T}}_{\text{YA,NWay}}\right)={\hat{F}}^{*}\left(T_{\text{Input,A,Classical}}\right)$, where $\hat{F}$ is calibrated using polynomial OLS regression, and ${\hat{F}}^{*}$ includes the calculation of ${\hat{T}}_{\text{YA,NWay}}$ from $T_{\text{Input,A,Classical}}$. The test set observations were then classified based on ${\hat{T}}_{\text{Qutput,A,Inverse}}$, according to the clusters found in the inverse metamodelling. See Additional file 1, sections S1.6 and S1.7 for a more comprehensive description of this methodology, including all predicting equations for test set observations.

QDA was chosen instead of LDA and Naive Bayes classification in this study, since LDA assumes the covariance matrix to be equal for all classes and Naive Bayes classification assumes that the presence of a particular feature of a class is unrelated to the presence of any other feature. In QDA, these assumptions are not made.

In the classical metamodelling, the sensitivity of each state variable to variations in the different parameters was estimated as the product of the second mode ***X***-factors and the transpose of the second mode ***Y***-factors (also called ***X-*** and ***Y-*** loadings), using the optimal number of NPLSR factors for the corresponding NPLSR model. The NPLSR loadings calculated here were not orthogonal. Work is currently in progress to assess the effects of using non-orthogonal loadings to estimate the model sensitivities.

### Additional sensitivity analyses

Some of the input-output relationships were not well described by the N-way HC-PLSR. Additional separate sensitivity analyses were therefore carried out for some of the state variables using 2-way second order polynomial HC-PLSR with the parameters and their cross-terms and second order terms as regressors and the state trajectories as response variables, analogous to the analysis presented in 8. The regressors were mean-centred and standardised prior to the HC-PLSR, while the state trajectories were only centred. Some of the state trajectories were logarithmised prior to the regression analysis.

The same clusters as in the N-way HC-PLSR described above were used. QDA 40 on predicted PLSR *Y-*scores (see Additional file 1, eq. S7d) were used for classification, and all PLS components (PCs) explaining a significant amount of the cross-validated *Y*-variance were included. Both in the global and regional regression analyses the optimal number of PLS components to use was found by 10-fold cross-validation. In the regional regression models, the matrices of cross-terms and second order terms were deflated with respect to the variation described by the first order terms (in an OLS regression) in order to better separate the effects of nonlinear terms and first order terms. The HC-PLSR was carried out in MATLAB® 41 Version 7.13 (R2011b), using in-house code that can be obtained from the authors upon request.

### Method benchmarking

For comparison, the inverse metamodelling was carried out using 2-way HC-PLSR where the 3-way state variable array was unfolded by concatenating the time series for all state variables, as well as by using aggregated outputs representing the state variable trajectories. The following aggregated outputs were derived from the state trajectories: period of oscillation, bottom, peak, time-to-bottom and time-to-peak for each state variable curve (see Additional file 1: Section S4). This resulted in 65 aggregated outputs. Both parameters, state variables and aggregated outputs were mean-centred and standardised prior to the HC-PLSR.

The number of PLS components to use in the PLSR models was chosen based on the percent explained cross-validated *Y*-variance as described in 8 and in the same way as for the N-way HC-PLSR, clustering and classification were done on the global *X-*scores of all PCs explaining a significant amount of the cross-validation variance. The same settings as described in 8 were used for the HC-PLSR, except that QDA 40 (the MATLAB® function "classify" from the Statistics Toolbox™ v7.6) was used for classification as in the N-way HC-PLSR. The same number of clusters as in the N-way HC-PLSR (6 clusters) was used also in the 2-way HC-PLSR analyses.

## Competing interests

The authors declare that they have no competing interests.

## Authors’ contributions

KT contributed to conception, wrote the MATLAB® code for the N-way HC-PLSR pipeline, performed the data analysis and wrote the paper. UGI participated in debugging of the N-way HC-PLSR code and in writing of the paper. ABG performed the computational experiments with the mammalian circadian clock model. SWO participated in writing the paper and HM contributed to conception and writing of the paper. All authors read and approved the final manuscript.

## Supplementary Material

Additional file 1 **S1. **Description of the multivariate metamodelling methodology; **S2. **Statistics of the global classical and inverse metamodels of the mammalian circadian clock model; **S3. **Supplementary sensitivity analyses of the mammalian circadian clock model; **S4. **Results from the method benchmarking. Additional file 1 includes references 5,7,8,11,19,20,21,22,23,29,34,35,36,37,38,40,41,42,43,44,45,46,47,48,49,50,51,52,53,54,55,56,57. Click here for file
