# Supplementary material for: Multi-way metamodelling facilitates insight into the complex input-output maps of nonlinear dynamic models
Source: BMC Syst Biol. 2012 Jul 20;6:88. doi: 10.1186/1752-0509-6-88 (PMC3483253; doi:10.1186/1752-0509-6-88)
Supplement: Additional file 1 — S1. Description of the multivariate metamodelling methodology; S2. Statistics of the global classical and inverse metamodels of the mammalian circadian clock model; S3. Supplementary sensitivity analyses of the mammalian circadian clock model; S4. Results from the method benchmarking. Additional file 1 includes references 578111920212223293435363738404142434445464748495051525354555657. [file 1752-0509-6-88-S1.pdf]

Supplementary material 1. Description of the multivariate metamodelling methodology

Hierarchical Cluster-based Partial Least Squares Regression (HC-PLSR) [1] is a generic method for multivariate metamodelling that yields compact, interpretable approximations of input-output relationships for complex, high-dimensional mathematical models, even when the models' input-output relationships are highly nonlinear. The N-way HC-PLSR provides this metamodelling functionality for models that yield N-way outputs, e.g. spatiotemporal data for a number of state variables obtained from simulations under various input conditions. The motive and mechanisms behind the N-way HC-PLSR method will here be outlined.

1.1 Motive and basic concepts

Models and metamodels

Mathematical models of complex systems tend to be high-dimensional and nonlinear, and the more detailed and realistic the mathematical models become, the more difficult the results are to overview and compute. Metamodelling can alleviate this, by providing both graphical overview and computational compaction (speed-up of computations).

Biological systems are often modelled by sets of coupled ordinary differential equations (ODEs), or for spatiotemporal models, by sets of coupled continuous partial differential equations (PDEs) and/or spatiotemporally discrete grid systems (Finite Elements). Such models have high-dimensional inputs and even higher-dimensional outputs, and usually give nonlinear input-output relationships. They call for multivariate metamodelling for computational compaction and behavioural overview, but the metamodelling must be able to handle unforeseen, abruptly nonlinear input-output relationships.

A mathematical model may be symbolised by the deterministic equation

$$Outputs=\boldsymbol{M}(Inputs) \qquad \text{(S0a)}$$

where $\boldsymbol{M}$ represents the mathematical model, e.g. a nonlinear ODE-based model. *Inputs* represents model parameters, initial conditions (e.g. initial state values) and/or various computational controls (e.g. integration accuracy required), while *Outputs* represents the outputs obtained from simulations with model $\boldsymbol{M}$. The outputs of interest could e.g. be time series of state variables and their spatial distributions, various quantities calculated from these state trajectories (e.g. time to peak of the curve, volume under the curve etc.), as well as computational performance ("cpu time to convergence").

With many *Inputs* and *Outputs* related through a complex nonlinear dynamic model $\boldsymbol{M}$, tools to overview, assess and simplify the use of this model are needed. Multivariate metamodelling is a handy tool for simplifying the use and construction of high-dimensional nonlinear mathematical models. Methodology for multivariate metamodelling will here be described and motivated, starting with simple linear and bilinear metamodelling tools and ending with the more powerful and versatile method N-way HC-PLSR introduced in this paper.

Classical and inverse metamodels

Based on *Inputs* and *Outputs* from computer simulations with model $\boldsymbol{M}$, two main types of multivariate metamodels of model $\boldsymbol{M}$ may be developed:
Classical ("causal") metamodels may be written

$$Outputs=\boldsymbol{C}(Inputs) + \Delta_C \qquad \text{(S0b)}$$

where function ***C(.)*** symbolises the ***C***lassical metamodel and Δ_C is the lack-of-fit between the *Outputs* generated by the actual model ***M*** and the approximations generated by the metamodel ***C(.)***. *Inverse* ("predictive") metamodels may be written

$$Inputs=\boldsymbol{I}(Outputs) + \Delta_I \qquad \text{(S0c)}$$

where function ***I(.)*** symbolises the ***I***nverse metamodel and Δ_I is the lack-of-fit between the *Inputs* to model ***M*** used in the simulations and the input values predicted from the *Outputs*, using metamodel ***I(.)***.

The classical and inverse metamodelling have different application arenas. Developed from the same set of simulation results, the two metamodel types *Outputs*=***C***(*Inputs*) and *Inputs*=***I*** (*Outputs*) reveal different aspects of the behaviour of the model ***M***. Therefore, in our opinion, the two methods should be used in parallel.

The classical metamodel *Outputs*=***C***(*Inputs*) shows how sensitive the *Outputs* are to changes and uncertainty in ***M***'s various *Inputs*, and can reveal possible "model sloppiness". The inverse metamodel *Inputs* =***I***(*Outputs*), in contrast, reveals possible covariation patterns - expected as well as unexpected - among the many *Outputs*, and can simplify parameterisation of models, and thereby provides a basis for future model improvement. Generally speaking, inverse metamodelling is superior to the classical metamodelling for finding how to split the metamodel into less complex, local sub-models in order to handle nonlinearities. Therefore, inverse metamodelling will be employed for optimising the nonlinearity-handling of the classical N-way HC-PLSR metamodelling (see section 1.7). In combination, the two metamodelling approaches make a powerful tool to analyse and describe the dynamic model behaviour, and in the following, we will describe how to combine classical and inverse metamodelling. First of all, metamodels are addressed in two distinct phases: Calibration and prediction.

"Calibration": Building the metamodel(s)

Based on *Inputs* and *Outputs* from a sufficiently comprehensive set of simulations, classical and inverse metamodels ***C(.)*** and ***I(.)*** can be developed by multivariate statistical data analysis. This training phase is here called "calibration". Like for the calibration of e.g. chemical or physical measuring instruments, this is simplified if the multivariate metamodelling method yields relatively simple, low-dimensional input-output descriptions suitable for graphical inspection in a relatively low number of plots, and include understandable but powerful validation tools to avoid over-parameterisation. What distinguishes metamodelling from e.g. calibration of instruments is that model ***M*** is often deterministic in the sense that the relationship *Outputs*=***M***(*Inputs*) has no noise - no random contributions apart from minor integrator errors etc. On the other hand, if the relationship *Outputs*=***M***(*Inputs*) is highly nonlinear, the metamodelling requires particular methods that give adequate handling of these nonlinearities. That is a main motive for the metamodelling method N-way HC-PLSR developed here.

"Prediction": Using the metamodel(s)

The metamodels ***C(.)*** and ***I(.)***, obtained from the calibration stage, may later be fitted to new data of the same general kind. Thereby, model output can be predicted for new combinations of input values, and new input values can be predicted from new sets of output data. The latter is useful for parameterising models using experimentally measured output data (see below).

Practical use of multivariate metamodelling

In short, multivariate metamodelling concerns how to *generate models of models*. This may e.g. be used for:

- *Providing insight:* overviewing how a nonlinear dynamic model behaves in practice, and how it can be simplified or improved through re-parameterisation. This was demonstrated for 2-way data in [1, 2], and is shown in the present paper for 3-way data. As described in [3], metamodelling can also be used to find biologically relevant parameter ranges and operative domains for dynamic models.
- *Computational compaction:* A successfully developed classical metamodel is useful for replacement of a slow computation $Outputs_{New}=\boldsymbol{M}(Inputs_{New})$ of the original model under new input conditions [4, 5]:

  $$Outputs_{New}=\boldsymbol{C}(Inputs_{New}) \qquad \text{(S0d)}$$

  This classical metamodel prediction gives accurate results, provided that the $Inputs_{New}$ are within the range of *Inputs* used in the calibration phase, that *Inputs* where sampled at sufficient density in this relevant range during calibration to allow local interpolation, and that the metamodelling method was indeed able to give an adequate description of the (nonlinear) *Input-Output* relationships.
- *Better fit to observations:* A successfully developed inverse metamodel may give a fast and robust estimation of unknown input parameters $Inputs_{New}$ from empirically measured outputs [4]. Provided that model $\boldsymbol{M}(.)$ is capable of generating $Outputs=\boldsymbol{M}(.)$ that can be related to those observed in $Outputs_{Measured}$, the inverse metamodel has the potential to yield fast predictions:

  $$Inputs_{New} =\boldsymbol{I}(Outputs_{Measured}) \qquad \text{(S0e)}$$

  This non-iterative prediction process can be a lot simpler than estimation of $Inputs_{New}$ by traditional iterative fitting of the nonlinear model $Outputs=\boldsymbol{M}(.)$ to data $Outputs_{Measured}$. Moreover, if several different input value combinations give equally good fit to the $Outputs_{Measured}$ data, e.g. the look-up approach described in [4] can be used to identify these. Finally, if model $\boldsymbol{M}(.)$ is not capable of generating outputs corresponding to $Outputs_{Measured}$, a combination of classical and inverse metamodelling can be used to identify outliers, as well as indicate how the model can be improved.
- *Model comparison:* Metamodelling can also be used for assessing the consequences of introducing changes to a mathematical model in a model construction process. New data ($Inputs_{New}$ and/or $Outputs_{New}$) may come from new input conditions for model $\boldsymbol{M}(.)$ or from simulations with comparable input conditions from another model to be compared to $\boldsymbol{M}(.)$. When both the new inputs and the new outputs are known, the new data may be fitted to both $\boldsymbol{C}(.)$ and $\boldsymbol{I}(.)$, for comparison with the old calibration data. The multivariate residuals $\boldsymbol{\Delta_C}$ and $\boldsymbol{\Delta_I}$ for the new simulations reveal the discrepancies (this approach is not pursued in the present paper).

1.2 The methodological background for the N-way HC-PLSR

Multivariate metamodelling of a model $Outputs=\boldsymbol{M}(Inputs)$ (eq. S0a) as used in e.g. [1, 2, 6] relies heavily on the use of vector- and matrix algebra to develop and combine regression-based metamodels of nonlinear dynamic systems. A tensor-based modelling approach that handles N-way output data will be described in detail below.

The *N-way Hierarachical Cluster-based Partial Least Squares Regression (N-way HC-PLSR) method* just to be described, combines various terms and successful data analytical tools

from the field of chemometrics, to be used in a different field – computational biology. The tools were originally developed for the study of complex chemical systems. The basic methodology and approaches of chemometrics, as e.g. described by Martens & Næs [7], has traditionally been used for comprehensive, but pragmatic explorative investigation and quantitative description. The causal structural or functional properties characteristic for data generated within computational biology represent a new area of applications for the chemometrics "toolbox" (that also shares several tools and approaches with computational statistics and machine learning). The strength of the chemometrics "toolbox" is its strong emphasis on real-world relevance, and on bringing the domain-specific knowledge of the system expert (chemist or biologist) into the data analysis via extensive graphics and prior knowledge.

Metaphorically, the bilinear data analysis tools used here can be considered as pragmatic information sieving devices –like fishermen's trawling nets – pulled by the explorer through an ocean of raw data - designed to catch the bigger fish while letting the small fry go. When working in complex systems that may be difficult to overview, the resulting data-driven models are only intended to represent the most desired catch, and the lack-of-fit residuals are summarised statistically. Once the overview has been obtained, more details can be pursued by more focused analysis, as demonstrated in the case study in Supplementary material 3.

The data driven models thus obtained can be regarded as compact, informative and potentially useful. However, they cannot be considered as *true*, neither in their structural form nor in parameter values. Although the linear model is followed by some laws of physics and chemistry, the estimated values of its many parameters are more or less meaningless individually. However, when applied together, they often provide accurate predictions and useful overviews. In combination with appropriate background knowledge, they facilitate access to meaningful results and corresponding insights into the studied system. This pragmatic cognitive orientation differs somewhat from traditional use of mathematical modelling in many fields of science. In e.g. physics, nonlinear dynamic models - their mathematical structures and their parameter values - are often intended to represent real phenomena – albeit in a simplified form.

1.3 Terminology and notation

Multivariate metamodelling concerns how to approximate the input-output relationships of a complex mathematical model ***M(.)*** by less computationally demanding data-driven statistical metamodels that are easier to overview and interpret. "Models of models" of course poses some terminology challenges, e.g. how to distinguish between the parameters in the original model ***M(.)*** and the statistical parameters of the metamodels. In the following, we have chosen to name the metamodel parameters by their technical descriptions (*means, scores, weights, loadings, regression coefficients, residuals* etc.), since they are generic, while the original model ***M(.)*** is characterised in terms of its *Inputs* and *Outputs*, specified in more detail when needed.

Moreover, to distinguish between the two directions in metamodelling, the classical modelling direction, $Outputs=\mathbf{C}(Inputs) + \Delta_C$, and the inverse direction, $Inputs=\mathbf{I}(Outputs) + \Delta_I$, are discussed in a common statistical regression framework, $Y=XB+F$, and subscripted for clarity when needed.

The choice of letters and symbols is a problem for interdisciplinary activities like metamodelling. In chemometrics, a pragmatic notation and terminology is normally used. A core of communally accepted symbols, *i, j, k, a, X, Y, T, W, P, Q, E* and *F* are often used, with only small local tradition nuances. In the present outline of the central chemometric data analysis methods that are the building blocks of the N-way HC-PLSR method, we stick to the notation tradition outlined in e.g. [7] for multivariate calibration, with some small modifications from path modelling [8] traditions required in order to extend this to N-way nonlinear multivariate metamodelling.

First, least-squares based regression analysis will be outlined briefly, along with subspace approximation by Principal Component Analysis (PCA) [9, 10], and combined into the multivariate

subspace regression method Principal Component Regression (PCR) [11]. Partial Least Squares Regression (PLSR) [7, 12–14], an extension of PCR developed to increase the *Y*-relevance of the estimated subspaces, will be motivated and summarised. Two extensions of the PLSR will be explained - N-way PLSR (NPLSR) [15, 16] to handle N-way arrays of simulation data, and Hierarchical Cluster-based PLSR (HC-PLSR) [1] to handle nonlinear subspace regression; these two are combined in the new N-way HC-PLSR developed here. Finally, the combined use of classical and inverse N-way HC-PLSR will be detailed, to allow quantitative metamodelling of highly nonlinear models ***M(.)***.

In data sets, variables (descriptors, attributes, observed quantities) are represented as columns, while observations (cases, samples, objects) are represented as rows. Here, scalars and one-way column vectors are denoted by lower-case characters in italics (e.g. x_{ik} or y_{ij}), two-way matrices as upper-case characters in italics (e.g. *X*), and N-way tensors by upper-case bold-face characters in italics. Indices $i=1,2,..,N$ represent observations (objects, cases, simulation conditions), $j=1,2,...,J$ and $k=1,2,...,K$ represent regressand variables *Y* and regressor variables *X*, respectively, while index $a=1,2,..,A$ represents abstract components (estimated latent variables/factors; see below). Predicted quantities are denoted using "hat" symbols (e.g. $\hat{Y}$).

1.4 Multivariate linear regression, principal component analysis and partial least squares regression

<u>The linear regression model</u>

The multiple linear regression model relates a regressand or response variable #*j*, y_j, to a set of *K* different regressor variables $X=[x_k, k=1,2,...,K]$ via a linear structure model with regression coefficient vector $b_j=[b_{kj}, k=1,2,...,K]$:

$$y_j = b_{0_j} + \sum_{k=1}^{K} x_k b_{k_j} + f_j = b_0 + Xb_j + f_j \qquad \text{(S1a)}$$

Here $y_j=[y_{ij}, i=1,2,...,N]$ and $X=[x_{ik}, i=1,2,...,N; k=1,2,...,K]$ represent different variables sorted so that their rows $i=1,2,...,N$ describe the same set of observations or samples; y_j is of dimension $(N \times 1)$, *X* is $(N \times K)$ and the lack-of-fit residual vector f_j is $(N \times 1)$.

For a set of *J* response variables $Y=[y_j, j=1,2,...,J]=[y_{ij}, i=1,2,...,N; j=1,2,...,J]$ simultaneously related to a common set of *K* regressor variables *X*, the corresponding multivariate linear regression model is given by:

$$Y = b_0 + XB + F \qquad \text{(S1b)}$$

where matrices *Y* and *F* are of dimension $(N \times J)$, and regression coefficient matrix *B* is $(K \times J)$.

<u>Data approximation, reliability balancing and linearisation by pre-processing</u>

The bilinear data analysis or modelling may be thought of as a generalised Taylor expansion of the – for us unknown – structure lying hidden within and between variable sets *X* and *Y* in the *N* available observations. In the metamodelling framework, this "hidden" structure is defined by the model, *Outputs*=***M***(*Inputs*). The bilinear model is therefore developed around the mean of each variable. In metamodelling, this ensures that good approximation is prioritised in the parameter region of most interest.

Linear and bilinear regressions employ ordinary least-squares (OLS) fit in various ways, something that requires balancing of the relevance and precision of the different data in *X* and *Y*. First of all, the present data analysis employs OLS across object *rows* $i=1,2...,N$, assuming that the *X*- and *Y*-data from the different observations (e.g. simulations) have approximately the same precision and relevance. If that is not the case, it is possible to replace the OLS by weighted or generalised least squares in each step of the methods to be presented here. Details on that is beyond

the scope of the present paper, since it usually suffices that irrelevant, peculiar or erroneous simulations are detected as *outliers* and kept out.

Moreover, in bilinear data analysis, the OLS principle is also used across *columns* $k=1,2,...,K$ in X and $j=1,2,...,J$ in Y. The resulting approximation model is therefore sensitive to the precision level of different variables within X and within Y used in the metamodel development. The user must therefore ensure that the scaling of the variables within X and within Y correspond to their expected or desired relevance and accuracy. Otherwise, the obtained bilinear model may be dominated by variables irrelevant for description of the analysed system due to large differences in absolute values or variances between the included variables.

Two different default scaling approaches are commonly used – either leaving the input variables in X and Y unchanged because they are all assumed to be equally precise and relevant, or standardising all variables to a standard deviation of one, giving them equal chance of contributing to - and being approximated by - the bilinear regression model. Thus, in the following description of the multivariate metamodelling methods, each of the variables X and Y will for simplicity be mean-centred. As mentioned, the variables can also be scaled to have comparable relevance/uncertainty as follows:

$$\bar{x}_k = \sum_{i=1}^{N} x_{ik} / N \qquad \bar{y}_j = \sum_{i=1}^{N} y_{ij} / N$$
$$x_{k,0} = (x_k - \bar{x}_k)/s_k \qquad y_{j,0} = (y_j - \bar{y}_j)/s_j \qquad \text{(S1c)}$$

where s_k and s_j represent the scaling factors that balance the levels of error or variance between the variables within X and within Y, e.g. the standard deviations over the N observations.

Likewise, it is presently assumed that the structure lying hidden within and between the present variable sets X and Y can be adequately approximated by a linearly or bilinearly additive model (sums and differences of a set of underlying variation phenomena). This means that types of variation not well represented by this additive modelling ought to be compensated for in a linearisation (pre-processing) stage. One example of this is when observations $i=1,2,...,N$ present "sideways" shifts between different X-variables $k=1,2,...,K$ in the fixed K-dimensional framework of a certain spatial representation, such as motion of observations in the K fixed pixel positions of a video camera or widely different temporal delays in a set of output time series. The additive approximation modelling is simplified if such phenomena are corrected for in a parameterised pre-processing, e.g. by motion estimation and motion compensation/warping [17]. However, non-additive pre-processing was not used in this paper.

It should be noted that a wide range of types of nonlinearities within and between the X- and Y-variables can be handled well by the local/regional bilinear data analysis approach to be presented below, and therefore do not necessitate cumbersome pre-processing. Therefore, with the present methodology, the user can choose to deal with e.g. highly nonlinear X-Y relationships and/or effects on Y of complicated interactions between X-variables in two alternative ways - either by increasing the complexity of the modelling sequence (first linearising in a pre-processing step, then using bilinear regression), or instead by increasing the complexity of the bilinear regression itself [1] (using polynomial bilinear models, combining several local bilinear regression models (each of which may be polynomial), or increasing the dimensionality of each bilinear model). Only the use of several local bilinear models and/or higher dimensionality of each bilinear model will be focused on here.

For mean-centred and scaled X-and Y-variables, the linear multivariate regression model eq. (S1a-b) becomes

$$Y_0 = X_0 B + F \qquad \text{(S1d)}$$

The linear regression model obtained via bilinear modelling

Different statistical methods may be used for estimating the regression coefficients *B* and the lack-of-fit residuals *F*. The choice depends on the assumptions made about the nature of the lack-of-fit residuals in *F* and about the relationships between the variables in *X* and in *Y*. Linear least-squares based regression methods are particularly versatile and simple, and are therefore used here.

Generally speaking, *least-squares* based regression methods make few, simple and natural assumptions: for instance, the lack-of-fit *Y*-residual elements f_{ij} are expected to be negative or positive with equal probability, so that their average is expected to be zero in the long run. Moreover, least-squares regression methods put the highest emphasise on reducing the *Y*-residual elements f_{ij} for observations *i* that have the most clearly distinguishable *X*-data (referred to as high "leverage"), i.e. with unique row vectors $x_i=[x_{ik}, k=1,2,\ldots,K]$ far from the mean of the other observations.

For residuals known to be independent, random and identically normally distributed, a more formal statistical motivation for the least squares methodology can also be made. In metamodel approximation of the input-output patterns of a deterministic model, many small nonlinear discrepancies between the model and its metamodel approximation may be expected to add up to apparently random, more or less normally distributed residuals, due to the central limit theorem. However, this statistical prerequisite is not necessary for the practical use of the principle of least squares, as long as subsequent statistical model validation and hypothesis testing is not based on rigid theoretical normality assumptions.

However, other assumptions may also be required, implicitly or explicitly, beyond these general least-squares assumptions. Different least-squares based regression methods make different additional assumptions, e.g. about possible patterns of collinearity between the *X*-variables (and to some extent also between the *Y*-variables). The traditional, full-rank OLS regression solution, often called Multiple Linear Regression (MLR), $B = (X_0{'}X_0)^{-1} X_0{'}Y_0$, assumes that *all the X-variables vary independently of each other*, so that $(X_0{'}X_0)$ can be inverted at full rank. This is often not the case, for instance if *X* represents model outputs, or inputs in an unbalanced subset of simulations from an initially balanced experimental design. Hence, estimation methods are required that can handle collinear regressors (and/or regressands).

The most traditional approach, stepwise regression, works technically but can give very misleading interpretations due to selection of non-causative, but correlated, variables instead of the causative variables. Statistical methods like e.g. ridge regression [18], Lasso [19] and Elastic nets [20] reduce this problem, but do not provide sufficient graphical insight into the different covariation patterns hidden in the data, at least not in their standard form. Reduced-rank subspace regression, which we focus on in this paper, offers both collinearity handling and good opportunities for graphical interpretation.

The two most common subspace regression approaches are the bilinear methods PCR [11] and PLSR [7, 12–14]. PCA [9, 10], on which PCR is based, is a well-known method in many fields of science, and PLSR is an extension of PCR. Therefore, the PCR will here be used as a backdrop for explaining and motivating the PLSR, which in turn is the basis for the Hierarchical Cluster-based PLSR [1] developed to handle nonlinearities, and its present N-way extension.

PCA, PCR and the bilinear regression model

In bilinear regression, the independence assumption is not applied for the *individual* variables in *X*, but instead on a *set of linear combinations* of the *X*-variables. These "super-variables" or *estimated latent X-variables* - the Principal Components– are here referred to as "score vectors from *X*" or

“PCs”, and denoted T_{XA}=[$t_{x,1}$,…, $t_{x,a}$,…, $t_{x,A}$], $A \leq K$. Each of the score vectors $t_{x,a}$ is defined as a weighted sum of the mean-centred X-variables,

$$t_{x,a} = \sum_{k=1}^{K} x_{k,0} v_{x,k,a} = X_0 * v_{x,a} \tag{S2a}$$

where each weight vector $v_{x,a}$ is of size (K x 1). For a set of $a=1,2,…,A$ PCs this can be written:

$$T_{XA} = X_0 V_{XA} \tag{S2b}$$

The sequence of A X-weight vectors V_{XA} =[$v_{x,1,…,}$ $v_{x,a,…,}$ $v_{x,A}$] is obtained by combining the X- and Y-data in a way that depends on prior knowledge about the system. For instance, in PCR, each consecutive weight vector $v_{x,a}$ is defined so that component $t_{x,a}$ explains as much of the remaining variation in X as possible; Y is ignored at this stage (which is identical to a PCA on X). Other bilinear regression methods, such as the PLSR and its nonlinear and N-way extensions; only differ in how the consecutive weight vectors $v_{x,k,a}$ are defined (see below).

In all these bilinear regression methods, these score vectors $t_{x,a}$, $a=1,2,…,A$ from X are, in turn, used for describing the pre-processed variables in Y according to the following model:

$$Y_0 = \sum_{a=1}^{A} t_{x,a} q_a' + F_A \tag{S3a}$$

This amounts to a multivariate linear regression of Y on T_{XA}:

$$Y_0 = T_{XA} Q_A' + F_A \tag{S3b}$$

The so-called *Y-loadings* Q (J x A) are estimated by OLS regression of Y_0 on T_{XA} , minimising, for each Y-variable, the sum of the squared Y-residuals F_A:

$$Q_A = Y_0' T_{XA} (T_{XA}' T_{XA})^{-1} \tag{S3c}$$

For more comprehensive graphical interpretation of the obtained regression solution, the pre-processed variables in X are likewise modelled in terms of their relationship to these estimated latent variables from X:

$$X_0 = \sum_{a=1}^{A} t_{x,a} p_a' + E_A \tag{S4a}$$

i.e. a linear regression of X on T_{XA}:

$$X_0 = T_{XA} P_A' + E_A \tag{S4b}$$

The OLS solution minimising, for each X-variable, the sum of the squared residuals in the X-residuals E_A is:

$$P_A = X_0' T_{XA} (T_{XA}' T_{XA})^{-1} \tag{S4c}$$

This reveals the reason why e.g. PCR is called a *bilinear* regression method: Eq. S4b shows that the set of X-variables is approximated by a so-called "bilinear" structure model $T_{XA}P_A'$, so named because it represents a linear model in both T_{XA} and P_A. Figure S1 illustrates the bilinear modelling of X, in terms of the vector expression in eq. S4a and the matrix expression in eq. S4b.

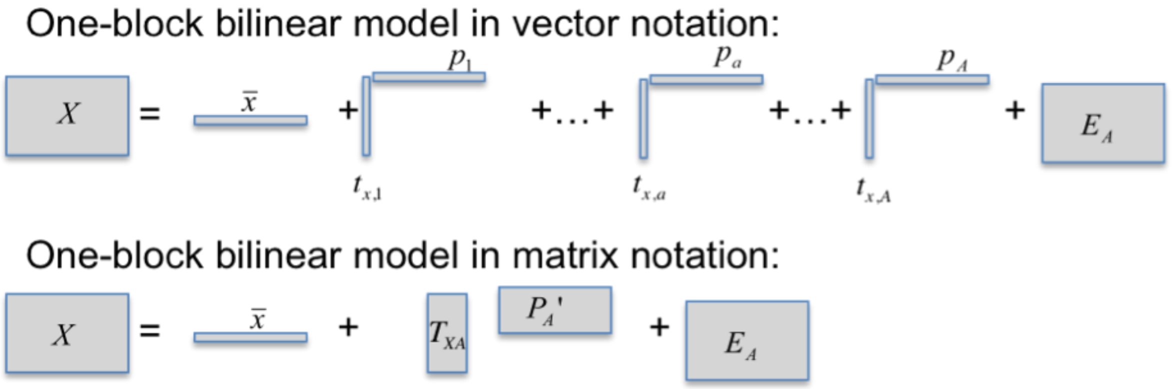

Figure S1. Illustration of bilinear models in vector and matrix notation.
Matrices are here illustrated as rectangles, while vectors are illustrated as lines.

An equivalent, single-step summary of this A-dimensional bilinear regression model in eqs. S3-S4 can be written in terms of the rank A linear regression model:

$$\hat{Y}_{0,A} = X_0 B_A' \quad \text{(S5a)}$$

where the (K x J) rank-A regression coefficient matrix B_A is defined by

$$B_A = V_{XA} Q_A' \quad \text{(S5b)}$$

<u>Optimising the complexity/dimensionality of the bilinear model</u>
The number of PCs in the bilinear regression model, A, reflects the number of independent variation phenomena in X that are Y-relevant. Said in another way, the PCs represent the A dimensions in the original K-dimensional X-space (spanned by the K X-variables) that are required in order predict Y optimally. In practice, one initially computes more PCs $a=1,2,.....A_{max}$ than *a priori* deemed necessary; afterwards the optimal number of PCs (A) is determined by some type of internal statistical validation, e.g. cross-validation, relegating the contributions of all subsequent PCs $A+1$, $A+2$,.....A_{max} to the residuals E_A and F_A.

Different systems for grouping how rows in X and Y are systematically kept out for testing during cross-validation allow the model to be validated according to different statistical criteria. In multivariate metamodelling, this can be used for testing the metamodel's ability to distinguish between different model versions, different model parameter combinations, different simulation conditions etc.

<u>Assessing the uncertainty of the obtained regression model parameters</u>
Summarising the model perturbations obtained during cross-validation gives pragmatic, so-called "jack-knife" estimates of how difficultly modelled variations in the X- and Y-data lead to uncertainty (inaccuracy or imprecision) in the resulting regression model. The jack-knife estimation of the variance/covariance of the linear model elements in B_A and b_{0A} is described in [14].

Graphical interpretation

The main patterns of sample similarities and differences are seen in plots of pairs or triplets of the first few columns in T_{XA} (called “score-plots”). The interpretation of these systematic patterns can be enhanced through plots of the corresponding columns of P_A and Q_A (called “loading-plots”). Graphical inspection of the columns # *j=1,2,…,J* in B_A and their uncertainty estimates reveals how the *X*-variables are combined in order to predict each of the *Y*-variables. Conversely, inspecting the rows # *k* =1,2,…,*K* in B_A and their uncertainty estimates shows how each *X*-variable # *k* is used for predicting the *J* different *Y*-variables. Unmodelled phenomena in *X* and *Y*, e.g unexpected outliers, can be seen in residual vectors e_{iA}, f_{iA} in matrices E_A, F_A, in combination with the observations’ so-called leverage $h_{i,A} = t_{x,i,A}(T_{XA}{}'T_{XA})^{-1}t_{x,i,A}{}'$, *i*=1,2,…,*N*.

Prediction: Applying the bilinear regression model to new observations

Bilinear prediction of Y_{New} from X_{New} in new observations, e.g. new inputs or new empirical measurements, requires the following steps: First, for each *X*-variable, pre-process the new observations by applying the previously computed mean-centering and scaling coefficients from the calibration phase (from eq. S1c):

$$x_{k,0,New} = (x_{k,New} - \bar{x}_k)/s_k$$
$$X_{0,New} = \lfloor x_{k,0,New}, k = 1,2,...,K \rfloor \qquad \text{(S6a)}$$

Then, based on the model’s previously estimated *X*-weights V_{XA}, predict the latent variables of the new observations:

$$T_{XA,New} = X_{0,New}V_{XA} \qquad \text{(S6b)}$$

Finally, multiply these with the model’s *Y*-loadings (eq. S6c) and add the model’s *Y*-mean and rescale (eq. S6d), to predict Y_{New} using *A* components:

$$\hat{Y}_{0,A,New} = T_{XA,New}Q_A{}' \qquad \text{(S6c)}$$

$$\hat{y}_j = \hat{y}_{0,j}s_j + \bar{y}_j,\ j = 1,2,..., J \qquad \text{(S6d)}$$

The *X*-residuals are obtained by

$$E_{New,A} = X_{0,New} - T_{XA,New}P_A{}' \qquad \text{(S6e)}$$

These residuals $E_{New,A}$ are important for evaluating the fit of the new observations to the obtained regression model, i.e. whether they are outliers (outside the object range calibrated for). In situations where this is unnecessary to check, an equivalent but faster prediction of Y_{New} from X_{New} is

$$\hat{Y}_{0,A,New} = X_{0,New}B_A \qquad \text{(S6f)}$$

followed by the inverse pre-processing (eq. S6d).

PLSR: an extension of PCR to ensure more *Y*-relevant *X*-components

As shown above, PCR consists of PCA of X_0 to estimate the *X*-weights V_{XA}, followed by OLS regression of *Y* on the PC scores $T_{XA}= X_0V_{XA}$. PLSR only differs from PCR in how the *X*-weights, V_{XA}, are defined: PCR yields V_{XA} purely based on information from *X*, while PLSR defines V_{XA} using both *X*- and *Y*-information. The PLSR is very similar to PCR, but merges the bilinear modelling of *X* and *Y* to maximise the amount of explained *X*-*Y* covariance. This is done by computing, in addition to the estimated latent *X*-variables $T_{XA} = \left[t_{x,1},...,t_{x,a},...,t_{x,A}\right]$ (eq. S2b), an auxillary set of latent *Y*-variables $T_{YA} = \left[t_{y,1},...,t_{y,a},...,t_{y,A}\right]$. These are defined by auxillary *Y*-weight vectors $V_{YA} = \left[v_{y,1},...,v_{y,a},...,v_{y,A}\right]$:

$$T_{YA} = Y_0V_{YA} \tag{S7a}$$

To maximise the total explained *X*-*Y* covariance, the PLSR yields values of the weight vectors $v_{x,a}$ and $v_{y,a}$ so that the covariance between $t_{x,a}$ and $t_{y,a}$ is maximised for each consecutive component $a=1,2,...,A$. Thereby, intercorrelation patterns between variables within *X* and intercorrelation patterns between variables within *Y* are used as valuable information that stabilises the regression of *Y* on *X*, in the sense that the *A* first few *X*-score vectors T_{XA}, used for modelling *Y*, have high *Y*-relevance.

After having been used in the iterative PLSR process defining *X*-weights V_{XA} and *X*-scores T_{XA}, the auxillary *Y*-weights V_{YA} and score vectors T_{YA} are only used for graphical interpretation; they are not part of the final bilinear regression model that predicts *Y* from *X* (eqs. S3-S6).

Equivalent PLSR implementations

The final prediction of the mean-centred and scaled *Y*-variables can thus be modelled from *X*. Two equivalent PLSR traditions exist. In one tradition, *Y* is modelled via predicted *Y*-scores $\hat{T}_{YA}$. An auxillary, inner relation model is then used in order to link the *X*-scores and the *Y*-scores:

$$T_{YA} = T_{XA}C_A + H_A \tag{S7b}$$

with the least- squares solution

$$C_A = (T_{XA}'T_{XA})^{-1}T_{XA}'T_{YA} \tag{S7c}$$

so that the predicted *Y*-scores are obtained as linear combinations of the *X*-scores:

$$\hat{T}_{YA} = T_{XA}C_A \tag{S7d}$$

and the *Y*-predictions become:

$$\hat{Y}_0 = \hat{T}_{YA}Q_A' \tag{S7e}$$

where the *Y*-loadings are estimated by the equivalent to eq. S3c:

$$Q_A = Y_0'\hat{T}_{YA}(\hat{T}_{YA}'\hat{T}_{YA})^{-1} \tag{S7f}$$

In the other tradition (see e.g. [7]), *Y* is modelled instead directly from the *X*-scores:

$$\hat{Y}_0 = T_{XA} Q_A' \qquad \text{(S7g)}$$

and the Y-loadings are estimated by the equivalent to eq. S3c:

$$Q_A = Y_0' T_{XA} (T_{XA}' T_{XA})^{-1} \qquad \text{(S7h)}$$

It should be noted that some authors use the term “PLS” to denote Partial Least Squares *regression*, while others use the same term for Partial Least Squares *path modelling* [8]. The former represents the purely data-driven, two-block multi-factor prediction method described above, while the latter represents a more theory-driven multi-block single-factor path modelling method. Hence, they represent two distinct methods and scientific traditions, even though they share Herman Wold’s original PLS concept of collecting maximal between-block covariance into a low-dimensional latent structure model of the relationships underlying the observed data. We here distinguish between the two traditions as is done in [7], and abbreviate Partial Least Squares regression by the noun “PLSR”. The shorter term “PLS” is only used as an adjective, qualifying and clarifying the nature of estimated quantities from PLSR, as in “PLS scores”, “PLS loadings” etc.

A number of equivalent numerical algorithms exist for PLSR. Usually, V_{XA} is obtained in terms of an orthonormal basis set of so-called loading weight vectors W_{XA}, from which V_{XA} can be computed:

$$V_{XA} = W_{XA} (P_{XA}' W_{XA})^{-1} \qquad \text{(S7i)}$$

This is done in order to ensure that the score vectors in T_{XA} are orthogonal to one another across the N samples ($T_{XA}' T_{XA}$ is diagonal) [13]. However, equivalent PLSR formulations with non-orthogonal score vectors T_{XA} also exist, in which the bilinear model is written $X_0 = T_{XA} W_{XA}' + E_A$ [7]. In the PLSR version used in the present paper (SIMPLS [21], with the function "plsregress.m" in MATLAB® [22] Statistics Toolbox™ v7.6), each of the auxillary Y-score vectors $t_{y,a}$, originally obtained by eq. S7a, are orthogonalised with respect to the a-1 previous score vectors in T_{XA} (i.e. $T_{X,a-1}$) so that ($T_{XA}' T_{YA}$) is lower triangular.

1.5 Local/regional bilinear modelling of highly nonlinear *X-Y* relationships

For a model ***M*** with highly nonlinear *Inputs-Outputs* relationships, the straight-forward linear or bilinear type of regression described above cannot give a metamodelling approximation of sufficient accuracy. For this, several nonlinear PLSR extensions are available.

One approach is to employ local linear modelling. It is well known that for a variable x and a variable y having a highly nonlinear relationship, a good regression approximation $y \approx f(x)$ can be obtained if the x-y regression analysis is performed in terms of a combined set of locally linear models. This principle is also useful in multivariate regression situations. If the X-Y relationship varies a lot from region to region in the score space T_{XA}, a highly nonlinear X-Y regression model is required, and the bilinear regression model is not applicable, in spite of its predictive and graphical advantages. However, if the score space is split into separate regions, each region can often be adequately described by the bilinear regression. This is the basis for Hierarchical Cluster-based Partial Least Squares Regression (HC-PLSR) [1], and is the approach used in the present paper.

A “global” X-Y PLSR model is first developed including all N observations (a polynomial PLSR may be used, as in [1], but for simplicity that is not pursued here). Then, based on the obtained PLS scores T_{XA} (or alternatively, T_{YA}), the N observations are grouped according to a cluster analysis. In our case we have chosen to use fuzzy C-means (FCM) clustering [23–26] for this purpose, since it allows the clusters to be partially overlapping, giving an estimated probability

for each observation to belong to the different clusters. For each cluster, a local PLSR model is then developed. The over-all model is optimised with respect to the number of local models required and to the number of PCs used in each local model.

An alternative approach is to split each individual input and/or output variable into a number (e.g. 10) of binary indicator variables. If the collection of these "qualitative" indicator variables are used in *X* or in *Y* in the metamodelling, abrupt, non-monotonous input-output curvatures can be handled well [27]. By including also their cross-products, rather complex interaction effects can also be detected.

Yet another approach is to extend the bilinear model to include explicit curvature. This can be done by replacing the linear *X*-*Y* mapping (eqs. S2b and S7a), by a nonlinear regression [28]. A simpler alternative is to extend the regressors *X* with new variables representing square terms and cross-products of the original *X*-variables (polynomial regression). This was combined with 2-way HC-PLSR in [1], but is skipped here, because it is more cumbersome for N-way data.

<u>Fuzzy clustering of simulations into more homogeneous subgroups</u>

As mentioned above, the N-way HC-PLSR employs locally linear metamodelling. This requires a segmentation and classification of the simulations into relatively homogeneous clusters suitable for linear regression. We have chosen fuzzy *C*-means (FCM) clustering [23–26] for this purpose, due to its flexibility.

In fuzzy cluster analysis a membership value u_{ic} is defined for each observation *i* and cluster *c*. The membership values are between 0 and 1, and must sum up to one for each observation *i*. In FCM the membership values are found by minimising

$$Mc = \sum_{c=1}^{C}\sum_{i=1}^{N} u_{ic}^{m} d_{ic}^{2}\,,\ m \geq 1 \qquad \text{subject to } \sum_{i=1}^{N} u_{ic} = 1 \qquad \text{(S8a)}$$

Here d_{ic} is the Euclidean distance between observation *i* and cluster *c* (i=1,2,…,N, c=1,2,…,C), *m* is a fuzzifier parameter that usually is set to be equal to 2.0. With m=1, FCM is the same as *K*-means clustering. *Mc* is minimised for a given $U=\{u_{ic}\}$ by setting the cluster centres v_c equal to the fuzzy means (see equation S8b). Next, the membership values that minimise *Mc* for given distances $D=\{d_{ic}\}$ are calculated using equation S8c. Then the *v*'s and the *d*'s are updated. This continues until convergence. The procedure is initialised randomly.

$$v_c = \frac{\sum_{i=1}^{N} u_{ic}^{m} x_i}{\sum_{i=1}^{N} u_{ic}^{m}} \qquad \text{(S8b)}$$

$$u_{ic} = \left(\sum_{k=1}^{C} \left(\frac{d_{ic}^{2}}{d_{ik}^{2}} \right)^{\frac{1}{m-1}} \right)^{-1} \qquad \text{(S8c)}$$

The basic FCM algorithm seeks spherical clusters. To find clusters with other shapes, modifications of the FCM algorithm must be applied, see for instance [29, 30].

1.6 N-way PCA (PARAFAC) and N-way Partial Least Squares Regression (NPLSR)

N-way extension of the bilinear data model for one data array ***X***

The data-driven modelling of a 2-way data matrix X (N x K), in terms of a bilinear decomposition of X after mean–centering the K variables, was in (eq. S4b) written: $X_0=T_{XA}P_A' + E_A$. In metamodelling, the outputs from the original dynamic model are often of "N-way" nature, e.g. 3-way arrays representing N simulation conditions x K_1 state variables x K_2 time points.

For data that is well described by a low-dimensional tensor model, the N-way or "N-linear" analogue to the bilinear data approximation model is the so-called *N-linear* structure model, which gives data approximation models that are simple, compact and interpretable. As illustrated in Figure S2, the N-way tensor model of a 3-way data array $\boldsymbol{X}$ (N x K_1 x K_2) may, after mean-centering each of the $K= K_1$ x K_2 variables, be written

$$\boldsymbol{X} = \bar{X} + \boldsymbol{T}_{XA,NWay}\boldsymbol{P}_{A,NWay}' + \boldsymbol{E}_{A,NWay} \quad \text{(S9a)}$$

for a model with A N-way "component tensors" or factors. These are defined by the set of A $\boldsymbol{X}$-weights $\boldsymbol{V}_{XA,NWay}$, which in turn, define the set of $\boldsymbol{X}$-score vectors, $\boldsymbol{T}_{XA,NWay}$:

$$\boldsymbol{T}_{XA,NWay} = \boldsymbol{X}_0\boldsymbol{V}_{XA,NWay} \quad \text{(S9b)}$$

The $\boldsymbol{X}$-weights $\boldsymbol{V}_{XA,NWay}$ are obtained by an iterative algorithm that, for a given number of factors, A, minimises a certain, method-dependent criterion - under the restriction that the $\boldsymbol{X}$-weight tensor for each component can be further decomposed into a bilinear model:

$$\boldsymbol{V}_{XA,NWay} = \left[v_{X,1}^{K1} \otimes v_{X,1}^{K2} \,|\ldots|\, v_{X,A}^{K1} \otimes v_{X,A}^{K2}\right] = \boldsymbol{v}_{X,A}^{K1} \circ \boldsymbol{v}_{X,A}^{K2} \quad \text{(S9c)}$$

Here $\circ$ denotes the Khatri-Rao product, $\boldsymbol{T}_{XA,NWay}$ is the $\boldsymbol{X}$-scores (first mode factors) and $\boldsymbol{v}_{X,A}^{K1}$ and $\boldsymbol{v}_{X,A}^{K2}$ are the loadings (or factors) for the second and third way/mode of $\boldsymbol{X}$, respectively.

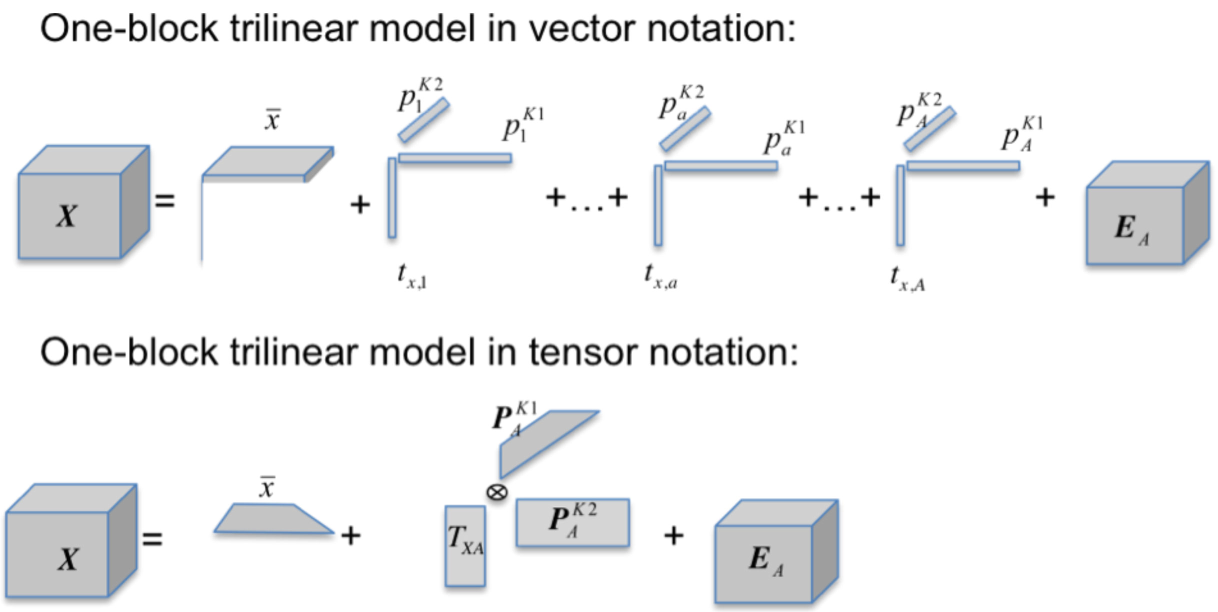

Figure S2. Illustration of trilinear models in vector and tensor notation.
Matrices are here illustrated as rectangles, vectors as lines and tensors as cubes.

PARAFAC (Parallel Factor Analysis) [31] is a prototype for N-linear data analysis, an N-way extension of PCA. In PARAFAC, the criterion to be minimised is the sum of squares in $\boldsymbol{E}_{A,Nway}$. In analogy to PCA, where the *X*-scores per definition may be equivalently obtained by $P_A=V_{XA}$ (eq. S2b), the PARAFAC model sets $\boldsymbol{P}_{A,NWay} = \boldsymbol{V}_{XA,NWay}$. Compared to the 2-way model (eq. S4b), the ***X***-loading tensor is

$$\boldsymbol{P}_{A,NWay} = \left[p_1^{K1} \otimes p_1^{K2} \mid \ldots \mid p_A^{K1} \otimes p_A^{K2} \right] = \boldsymbol{P}_A^{K1} \circ \boldsymbol{P}_A^{K2} \qquad \text{(S9d)}$$

where $\circ$ denotes the Khatri-Rao product, $\boldsymbol{T}_{XA,NWay}$ is the ***X***-scores and $\boldsymbol{P}_A^{K1}$ and $\boldsymbol{P}_A^{K2}$ are the loadings for the second and third mode of ***X***, respectively. PARAFAC is not used in this paper; like PCA it does not involve the *Y*-variables. In addition, it converges relatively slowly. Instead, an N-way extension of PLSR is used here.

<u>N-way extension of PLSR: NPLSR</u>

The N-way analogue to PLS regression (NPLSR) to be used here [15, 16] is defined in equations S9a-S13 for 3-way data. Again, the decomposition of ***X*** is given by eq. S9a. For an NPLSR model with *A* components, the ***X***-weights $\boldsymbol{V}_{XA,NWay}$ are determined by an iterative PLS-procedure so as to maximise the covariance between the ***X***-scores $\boldsymbol{T}_{XA,NWay}$ (eq. S9b) and a corresponding matrix of auxillary ***Y***-scores $\boldsymbol{T}_{YA,NWay}$. This process also defines $\boldsymbol{V}_{YA,NWay}$. In summary, the iterative estimation process employs a temporary model for the decomposition of ***Y***:

$$\boldsymbol{Y}_0 = \boldsymbol{T}_{YA,NWay} \boldsymbol{V}_{YA,NWay}' + \boldsymbol{F}_{A,NWay} \qquad \text{(S10a)}$$

where $\boldsymbol{T}_{YA,NWay}$ and $\boldsymbol{V}_{YA,NWay}$ are the ***Y***-scores and ***Y***-weights, respectively, with

$$\boldsymbol{T}_{YA,NWay} = \boldsymbol{Y}_0 \boldsymbol{V}_{YA,NWay} \qquad \text{(S10b)}$$

This temporary ***Y***-model is only used during the iterative process that leads to the estimation of $\boldsymbol{V}_{YA,NWay}$ and for graphical interpretation; it is not part of the final NPLSR model. $\boldsymbol{E}_{A,NWay}$ and $\boldsymbol{F}_{A,NWay}$ denote the residuals in ***X*** and ***Y***, respectively, after having subtracted the *A* NPLSR factors. In the version of NPLSR used here, ***X*** may be N-way, ***Y*** may be N-way, or both.

The ***X***-loadings in the NPLSR are, upon convergence, simply defined as the ***X***-weights

$$\boldsymbol{P}_{A,NWay} = \boldsymbol{V}_{XA,NWay} \qquad \text{(S11)}$$

The final prediction of the mean-centred and scaled ***Y***-variables can thus be modelled from ***X***. Equivalent to eq. S7, there are two equivalent modelling conventions possible for the NPLSR. In one convention, ***Y*** is modelled via predicted ***Y***-scores $\hat{\boldsymbol{T}}_{YA,NWay}$. An auxillary inner relation model is then used in order to link the ***X***-scores and the ***Y***-scores:

$$\boldsymbol{T}_{YA,NWay} = \boldsymbol{T}_{XA,NWay} \boldsymbol{C}_A + \boldsymbol{H}_A \qquad \text{(S12a)}$$

with the least- squares solution

$$\boldsymbol{C}_A = (\boldsymbol{T}_{XA,NWay}' \boldsymbol{T}_{XA,NWay})^{-1} \boldsymbol{T}_{XA,NWay}' \boldsymbol{T}_{YA,NWay} \qquad \text{(S12b)}$$

so that the predicted ***Y***-scores are obtained as linear combinations of the ***X***-scores:

$$\hat{\boldsymbol{T}}_{YA,NWay} = \boldsymbol{T}_{XA,NWay}\boldsymbol{C}_{\boldsymbol{A}} \quad \text{(S12c)}$$

Hence, the ***Y***-predictions become:

$$\hat{\boldsymbol{Y}}_0 = \hat{\boldsymbol{T}}_{YA,NWay}\boldsymbol{Q}_{A,NWay}' \quad \text{(S13a)}$$

where the ***Y***-loadings are estimated by the equivalent to eq. S3c:

$$\boldsymbol{Q}_{A,Nway} = \boldsymbol{Y_0}'\hat{\boldsymbol{T}}_{YA,Nway}(\hat{\boldsymbol{T}}_{YA,Nway}'\hat{\boldsymbol{T}}_{YA,Nway})^{-1} \quad \text{(S13b)}$$

In the other convention, ***Y*** is modelled instead directly from the ***X***-scores:

$$\hat{\boldsymbol{Y}}_0 = \boldsymbol{T}_{XA,NWay}\boldsymbol{Q}_{A,NWay}' \quad \text{(S13c)}$$

and the ***Y***-loadings are estimated by the equivalent to eq. S3c:

$$\boldsymbol{Q}_{A,Nway} = \boldsymbol{Y_0}'\boldsymbol{T}_{XA,Nway}(\boldsymbol{T}_{XA,Nway}'\boldsymbol{T}_{XA,Nway})^{-1} \quad \text{(S13d)}$$

The *A* NPLSR factors possess many of the same properties as the PCs from 2-way PLSR. However, they are not orthogonal, and therefore one cannot estimate one component at a time. To optimise the model complexity, a range of full NPLSR models, with *A=1,2,....,Amax* factors, are fitted to the ***X-Y*** data, and assessed for predictive validity e.g. by cross-validation, and the lowest number of factors giving sufficient predictive ability is chosen as the final model rank, *A*.

1.7 N-way Hierarchical Cluster-based PLS regression (N-way HC-PLSR)

<u>Motivation</u>

The purpose of the N-way HC-PLSR is to provide prediction models $\boldsymbol{Y} \approx f(\boldsymbol{X})$ from data ***X*** and ***Y*** in a training set of *N* “calibration observations”, in a situation where ***X*** and/or ***Y*** are N-way arrays, and the ***X-Y*** relationship is expected to be highly nonlinear, i.e. the relationship between ***Y*** and ***X*** varies strongly between different regions of the ***X***-space.

The need for the new N-way HC-PLSR method for e.g. multivariate metamodelling is based on the following: The statistical prediction model $\boldsymbol{Y} \approx f(\boldsymbol{X})$ is to be used for a range of different types of *Inputs* and *Outputs* for model ***M(.)***; therefore the metamodelling tool must be rather versatile. Since it is to be used for interpretation through graphical inspection of the main relationship patterns within and between ***X*** and ***Y***, the solution should be of low rank to facilitate graphical displays. The solution is also to be used for quantitative prediction of ***Y*** from ***X***, both in the cross-validation sense for rank optimisation, and for prediction of ***Y*** from ***X*** for new observations. Hence, it should be statistically parsimonious to avoid over-fitting, and it must provide adequate handling of ***X-Y*** nonlinearities, which is here attained by a hierarchy of locally bi- or trilinear models.

All the building blocks making up the N-way HC-PLSR have been described above. Here they are all combined to generate a generic metamodelling tool. Below, the Classical and Inverse metamodelling procedures used in this paper are expressed in explicit algebra.

Classical and inverse metamodelling

As described in section 1.1, the data-driven metamodelling can be used in two different ways: Classical and Inverse metamodelling. This is the case for both the PLSR, NPLSR, HC-PLSR and N-way HC-PLSR metamodelling. While the PLSR and NPLSR are suitable for models ***M(.)*** with reasonably linear, or at least monotonous *Inputs* - *Outputs* relationships, the HC-PLSR and N-way HC-PLSR can also handle abruptly nonlinear *Inputs* - *Outputs* relationships. However, for successful classical metamodelling using N-way HC-PLSR, we have found it advantageous to use an intermediate inverse metamodel to ensure meaningful separation of the observations into local sets used for local regression analysis. In the following we give a more detailed description of the N-way HC-PLSR procedure, as used for inverse and classical metamodelling.

Figure 2 in the main manuscript gives an overview of the most essential steps of the inverse (top) and classical (bottom) metamodelling, both in the calibration (training) phase (left) and the prediction (future use) phase (right). It also outlines how the inverse metamodelling can be used for improved handling of nonlinearities in the classical metamodelling by facilitating meaningful inputs to the cluster analysis.

For data obtained from computer simulations with a nonlinear model, a clear causal direction is evident: its outputs are caused by its inputs: *Outputs*=***M***(*Inputs*) (eq. S0a), and not the other way around. It may thus be tempting to start with generating a metamodel of the nonlinear model in the same way, with ***X*** =*Inputs* and ***Y***=*Outputs* in a classical N-way metamodel ***Y***=***C***(***X***) + $\boldsymbol{\Delta}_C$ (eq. S0b). This type of metamodelling is indeed useful for sensitivity analysis, as well as for computational compaction, since it allows fast prediction of *Outputs* from *Inputs* under new conditions. However, the N-way HC-PLSR may require an initial inverse metamodelling step in order to work optimally. This discussion therefore starts with the inverse metamodelling.

Inverse metamodelling by N-way HC-PLSR

Calibration (Fig. 2 (main manuscript)- top left): For models ***M*** with N-way *Outputs* and highly nonlinear *Inputs* – *Outputs* relationships, inverse metamodelling can be attained by straight-forward application of the N-way HC-PLSR. During calibration, a rough, global over-all metamodel is first developed, to be used for cluster analysis: The global ***X***-weights $\boldsymbol{V}_{XA,NWay}$are estimated by the NPLSR algorithm, along with the ***X***-scores $\boldsymbol{T}_{XA,NWay}$. These ***X***-scores, which are linear combinations of the *Outputs* from ***M(.)***, are used for splitting the set of *N* simulation conditions into more homogeneous subgroups – in our case by fuzzy clustering (eq. S8). Then, within each of these clusters, a local inverse NPLSR metamodel is developed.

Prediction: (Fig. 2 (main manuscript)- top right): New *Inputs* can be predicted from new *Outputs* by applying eq. S13, after a classification on predicted ***X***-scores (from eq. S9b) and a selection of the appropriate local NPLSR model for each new observation. We use Quadratic Discriminant Analysis (QDA) [32] for classification in the present work.

Classical metamodelling by N-way HC-PLSR

Calibration (Fig. 2 (main manuscript)- bottom left): While the input parameters are usually easy to control in computer simulations, they do not in themselves tell much about the model ***M(.)*** behaviour. In contrast, the outputs form a better basis for clustering the simulations into local regions with less complex input-output relationships. The output information needed for the clustering can be summarised by the *Outputs*-scores $\boldsymbol{T}_{Output,A}$, which can be obtained in several ways. Useful estimates might be obtained directly during the classical metamodelling from the temporary ***Y***-scores, as $\boldsymbol{T}_{Output,A} = \boldsymbol{T}_{YA,NWay}$ (from eq. S10b). However, we have instead chosen to estimate the *Outputs*-scores from an inverse metamodel on the same data, since a PLSR model is asymmetric- it is defined primarily based on the ***X***-scores $\boldsymbol{T}_{XA,Nway}$, not the ***Y***-scores. The inverse

modelling direction, in which *Outputs* =$\boldsymbol{X}$, therefore gives more information about unexpected patterns among the model *Outputs*, and thus about how the input-output relationship differs under different *Inputs* conditions. Hence, we define $\boldsymbol{T}_{Output,A} = \boldsymbol{T}_{Output,A,Inverse} = \boldsymbol{T}_{XA,NWay}$(from eq. S9b) from the inverse metamodel in which $\boldsymbol{X}$ = *Outputs* and $\boldsymbol{Y}$= *Inputs*.

Still, to be able to quantify $\boldsymbol{T}_{Output,A,Inverse}$ in future situations in which unknown *Outputs* are to be predicted from known *Inputs*, we need to obtain the most informative scores from the scores of the known *Inputs*, which are obtained in the classical metamodelling as $\boldsymbol{T}_{XA,NWay}$. Hence, we relate the metamodelling scores $\boldsymbol{T}_{XA,Nway}$ from the inverse metamodelling to the metamodelling scores $\boldsymbol{T}_{XA,Nway}$ from the classical metamodelling during calibration as follows: With $\boldsymbol{T}_{OutputA,Inverse} = \boldsymbol{T}_{XA,NWay}$ from the inverse metamodel and $\boldsymbol{T}_{InputA,Classical} = \boldsymbol{T}_{XA,NWay}$ from the classical metamodel, a second-degree polynomial OLS regression model is employed in order to ensure a reasonable approximation of possible input-output nonlinearities. This polynomial conversion model is here symbolised by function *F(.)*:

$$\boldsymbol{T}_{OutputA,Inverse} = F^{*}(\boldsymbol{T}_{InputA,Classical}) + \Delta \qquad \text{(S14a)}$$

with the OLS solution abbreviated as $\hat{F}(.)$:

$$\hat{\boldsymbol{T}}_{OutputA,Inverse} = \hat{F}^{*}(\boldsymbol{T}_{InputA,Classical}) = \hat{F}(\hat{\boldsymbol{T}}_{YA,NWay}) \qquad \text{(S14b)}$$

In this work, $\hat{\boldsymbol{T}}_{YA,NWay}$ was used to estimate the function *F(.)*, but according to eq. S12c, $\hat{\boldsymbol{T}}_{YA,NWay}$is a linear combination of $\boldsymbol{T}_{XA,NWay}$, making this an implicit part of $\hat{F}^{*}(.)$. The different simulations in the calibration set are split into the same local clusters as used in the inverse metamodelling, and separate classical metamodels are finally developed within each cluster.

Prediction (Fig. 2 (main manuscript)- bottom right): New inputs are defined as $\boldsymbol{X}_{\mathrm{New}}$ and projected into the classical metamodel for prediction of input scores $\boldsymbol{T}_{XA,NWay,New}$ (eq. S9b). Defining $\boldsymbol{T}_{InputA,Classical,New} = \boldsymbol{T}_{XA,NWay,New}$, we obtain $\hat{\boldsymbol{T}}_{OutputA,Inverse,New} = \hat{F}^{*}(\boldsymbol{T}_{InputA,Classical,New})$. From $\hat{\boldsymbol{T}}_{OutputA,Inverse,New}$, we can now classify each new object with respect to the different calibration clusters (here done using QDA). Once classified into one or more local calibration classes, the appropriate local classical calibration model is employed for prediction of the unknown $Outputs_{New}$ from the known $Inputs_{New}$.

1.8 Concluding remarks

The toolbox of methods for multivariate metamodelling, leading up to the N-way HC-PLSR, has here been described and motivated. The way we have combined the tools is not necessarily the optimal; a number of tools in the toolbox may well be replaced by other tools. However, the metamodelling methodology needs to address the following basic topics: the collinearity among *Outputs* and sometimes even among *Inputs;* the need for low-dimensional graphical model summaries; the importance of validation tools to avoid over-parameterisation of the metamodels; the need for good predictive ability, both for classical and for inverse metamodelling; the need for methods that handle highly nonlinear *Inputs-Outputs* relations, and the need for methods that handle N-way *Inputs* and/or *Outputs.*

1.9 References

1. Tøndel K, Indahl UG, Gjuvsland AB, Vik JO, Hunter P, Omholt SW, Martens H: **Hierarchical Cluster-based Partial Least Squares Regression is an efficient tool for metamodelling of nonlinear dynamic models**. *BMC Syst Biol* 2011, **5**:90.

2. Martens H, Veflingstad S, Plahte E, Martens M, Bertrand D, Omholt S: **The genotype-phenotype relationship in multicellular pattern-generating models - the neglected role of pattern descriptors**. *BMC Syst Biol* 2009, **3**:87.

3. Tøndel K, Gjuvsland AB, Måge I, Martens H: **Screening design for computer experiments: Metamodelling of a deterministic mathematical model of the mammalian circadian clock**. *J Chemometr* 2010, **24**:738–747.

4. Isaeva J, Sæbo S, Wyller JA, Nhek S, Martens H: **Fast and comprehensive fitting of complex mathematical models to massive amounts of empirical data**. *Chemometr Intell Lab*, **In press**.

5. Isaeva J, Martens M, Sæbø S, Wyller JA, Martens H: **The modelome of line curvature: Many nonlinear models approximated by a single bi-linear metamodel with verbal profiling**. *Physica D* 2012, **241**:877–889.

6. Kleijnen JPC: *Design and Analysis of Simulation Experiments*. 1st edition. New York, USA: Springer; 2007.

7. Martens H, Næs T: *Multivariate calibration*. Chichester, UK: John Wiley and Sons; 1989.

8. Tenenhaus M, Vinzi VE, Chatelin Y-M, Lauro C: **PLS path modeling**. *Comput Stat Data Anal* 2005, **48**:159–205.

9. Pearson K: **On lines and planes of closest fit to systems of points in space**. *Philos Mag* 1901, **2**:559–572.

10. Jolliffe IT: *Principal Component Analysis*. 2nd edition. Springer; 2002.

11. Jolliffe IT: **A Note on the Use of Principal Components in Regression**. *J Roy Stat Soc C-App* 1982, **31**:300–303.

12. Krishnaiah PR: *Multivariate Analysis*. Academic Press Inc; 1967.

13. Wold S, Martens H, Wold H: **The multivariate calibration method in chemistry solved by the PLS method.** In *Lecture notes in Mathematics, Matrix Pencils*. Heidelberg: Springer-Verlag; 1983:286–293.

14. Martens H, Martens M: *Multivariate Analysis of Quality: An Introduction*. 1st edition. Wiley; 2001.

15. Bro R: **Multiway calibration. Multilinear PLS**. *J Chemometr* 1996, **10**:47–61.

16. Andersson CA, Bro R: **The N-way Toolbox for MATLAB**. *Chemometr Intell Lab* 2000, **52**:1–4.

17. Westad F, Martens H: **Shift and intensity modeling in spectroscopy--general concept and applications**. *Chemometr Intell Lab* 1999, **45**:361–370.

18. Hoerl AE, Kennard RW: **Ridge Regression: Biased Estimation for Nonorthogonal Problems**. *Technometrics* 1970, **12**:55–67.

19. Tibshirani R: **Regression Shrinkage and Selection via the Lasso**. *J Roy Stat Soc B Met* 1996, **58**:267–288.

20. Zou H, Hastie T: **Regularization and variable selection via the Elastic Net**. *J Roy Stat Soc B* 2005, **67**:301–320.

21. de Jong S: **SIMPLS: An alternative approach to partial least squares regression**. *Chemometr Intell Lab* 1993, **18**:251–263.

22. *MATLAB®, v. 7.13*. The MathWorks™; 2011.

23. Bezdek JC: *Pattern Recognition with Fuzzy Objective Function Algorithms*. Kluwer Academic Publishers; 1981.

24. Berget I, Mevik B-H, Næs T: **New modifications and applications of fuzzy C-means methodology**. *Comput Stat Data Anal* 2008, **52**:2403–2418.

25. Næs T, Isaksson T: **Splitting of calibration data by cluster analysis**. *J Chemometr* 1991, **5**:49–65.

26. Næs T, Kubberød E, Sivertsen H: **Identifying and interpreting market segments using conjoint analysis**. *Food Qual Prefer* 2001, **12**:133–143.

27. Martens, Harald: **Non-linear multivariate dynamics modelled by PLSR**. In *Proceedings of the 6th International Conference on Partial Least Squares and Related Methods*. Beijing: Publishing House of Electronics Industry; 2009:139–144.

28. Berglund A, Wold S: **INLR, implicit non-linear latent variable regression**. *J Chemometr* 1997, **11**:141–156.

29. Gath I, Geva AB: **Unsupervised Optimal Fuzzy Clustering**. *IEEE Trans Pattern Anal Mach Intell* 1989, **11**:773–780.

30. Frigui H, Krishnapuram R: **A robust competitive clustering algorithm with applications in computer vision**. *IEEE Trans Pattern Anal Mach Intell* 1999, **21**:450–465.

31. Bro R: **PARAFAC. Tutorial and applications**. *Chemometr Intell Lab* 1997, **38**:149–171.

32. Hastie T, Tibshirani R, Friedman JH: *The Elements of Statistical Learning*. Corrected. Springer; 2003.

Supplementary material 2. Statistics of the global classical and inverse metamodels of the mammalian circadian clock model

2.1 Explained cross-validated *X*- and *Y*-variance

In the inverse metamodelling, the 3-way state variable trajectory array was used as regressor (***X***) and the parameters were used as response variables (*Y*). A sequence of *Y*-relevant tensor products (NPLS factors) were generated from the N-way ***X***-array, as described in Supplementary material 1, and used for prediction of the 2-way parameter combination data, inverting the causal direction from input parameters to model outputs. When applied to the data from the whole parameter range tested, this inverse NPLS regression indicated that 19 NPLS factors were needed, altogether explaining 50% of the cross-validated calibration set variance in the model inputs (Figure S3A).

Conversely, in the classical metamodelling, the parameters were used as regressors (*X*) to predict the state variable trajectories (***Y***), and a sequence of ***Y***-relevant factors were generated from the matrix of model input parameters to predict the state variable trajectories in the N-way model output array ***Y***. Based on Figure S3B, using 8 factors in the global classical NPLSR model was considered optimal, explaining 54.3% of the cross-validated calibration set variance in the state trajectories ***Y***.

The dissimilarity in metamodel rank between the inverse and classical predictions reflected the difference in data structure between the input parameter- and model output data.

A B

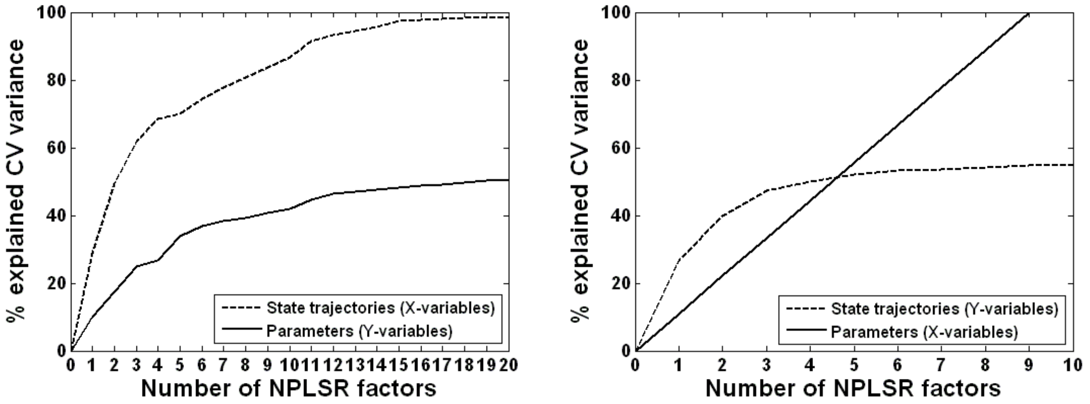

Figure S3. Results from the calibration of the inverse and classical global NPLSR metamodels.

A) Explained cross-validated ***X***- and *Y*-variance from the global inverse metamodel. Using 19 NPLSR factors was considered optimal.

B) Explained cross-validated *X*- and ***Y***-variance from the global classical metamodel. Using 8 NPLSR factors was considered optimal.

Supplementary material 3. Supplementary sensitivity analyses of the mammalian circadian clock model

In order to obtain insight into the relationships between all varied input parameters and all the mammalian circadian clock state variables, separate sensitivity analyses with 2-way HC-PLSR were carried out for the state variables that were not described sufficiently well by the N-way HC-PLSR (which included all state trajectories simultaneously).

3.1 Separate sensitivity analysis for the state variable I_N

The global second order polynomial PLSR model (from the HC-PLSR) obtained using the parameters and their cross-terms and second order terms as regressors, and the logarithm of the I_N state trajectory (concentration of inactive complex between Per-Cry and Clock-Bmal1 in the nucleus) as response gave a mean R^2-value over the trajectory of 0.94 in the test set prediction. There was no gain from using regional analysis of the input-output relationships for this state variable, and the regression coefficients for the 200 time steps in the trajectory from the global PLSR were therefore used as sensitivity measures (Figure S4). The fact that this state variable had to be logarithmised prior to the analysis may explain why it could not be described together with the other state variables in the N-way HC-PLSR metamodel. As shown in Figure S4, the parameters v_{mB}, v_{mC}, v_{mP}, v_{dIN}, k_1 and k_7 had the largest effects on the I_N state trajectory behaviour. Several interactions between these parameters were also identified (Figure S4B).

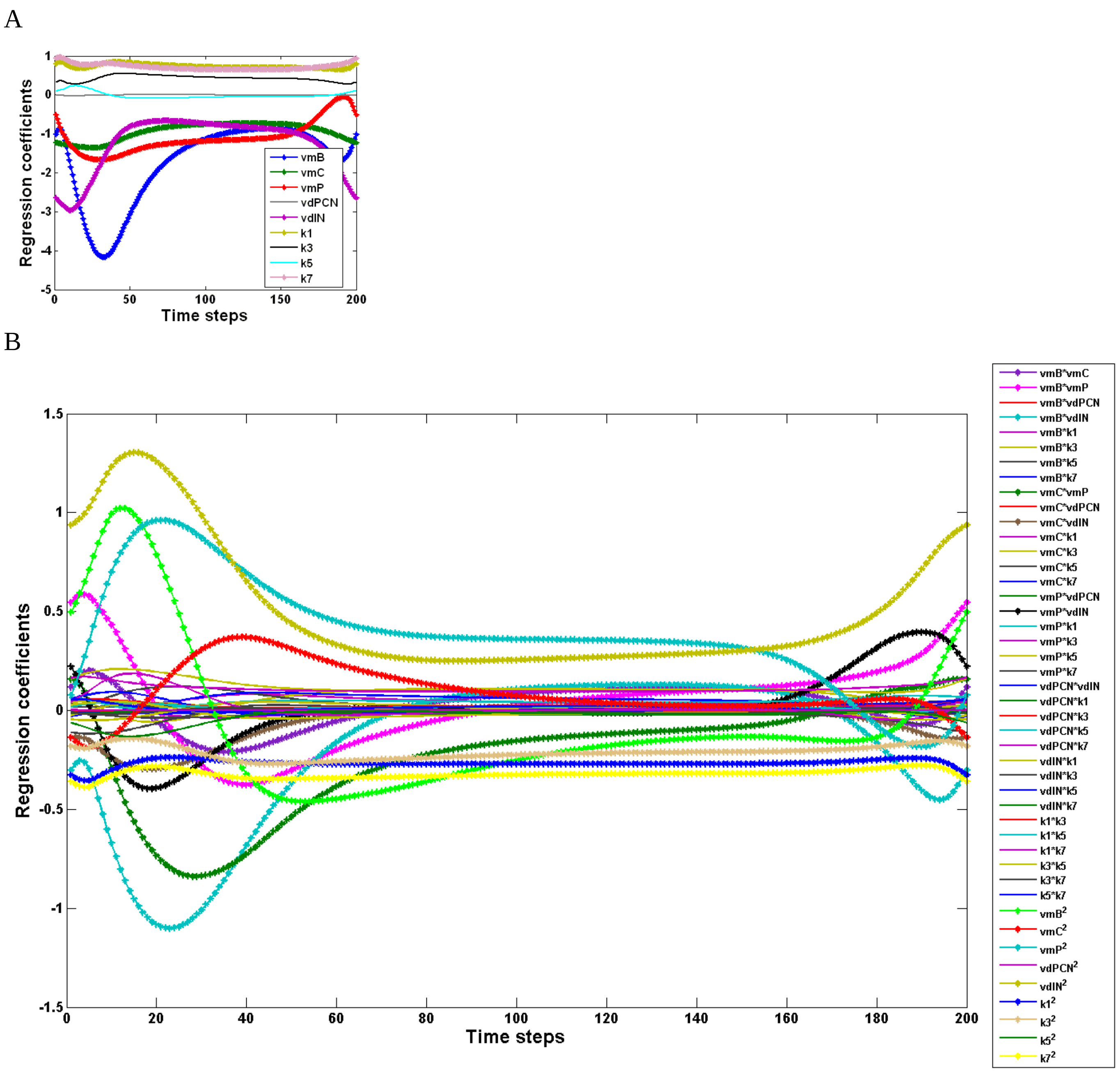

Figure S4. Results from the sensitivity analysis of the state variable I_N.
Regression coefficients for A) the main effects of the circadian clock input parameters and B) the cross-terms and second order terms of the input parameters from the global PLSR-based sensitivity analysis of the state variable I_N. The variables showing the largest effects on the state trajectory are marked with a star symbol.

3.2 Separate sensitivity analysis for the state variable B_N

The global second order polynomial PLSR model obtained using the parameters and their cross-terms and second order terms as regressors and the B_N state trajectory (concentration of non-phosphorylated Bmal1 protein in the nucleus) as response gave a mean R^2-value over the trajectory of 0.93 in the test set prediction. There was only 2% gain in mean test set prediction R^2 from using regional analysis, but both the global and regional regression coefficients from the HC-PLSR for the 200 time steps in the trajectory were still analysed (Figure S5 and S6). The results from the global PLSR-based sensitivity analysis shown in Figure S5 indicated that the state variable B_N was

most sensitive to the input parameters v_{mB} and v_{mP}, and that there was an interaction between these two parameters and between v_{mB} and k_5.

A

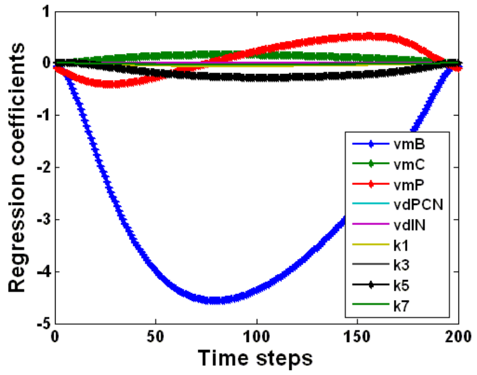

B

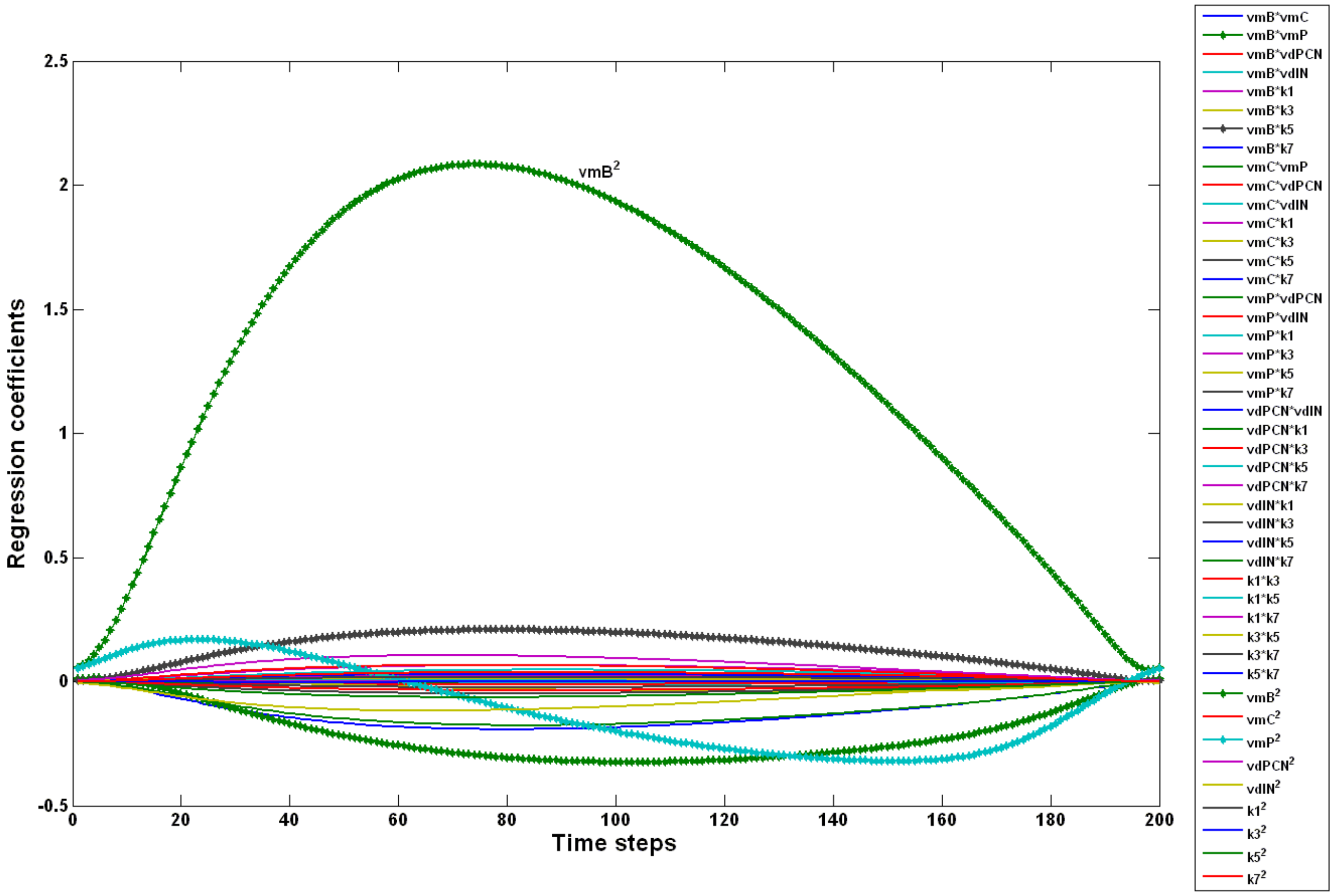

Figure S5. Results from the global sensitivity analysis of the state variable B_N. Regression coefficients for A) the main effects of the circadian clock input parameters and B) the cross-terms and second order terms of the input parameters from the global PLSR-based sensitivity analysis of the state variable B_N. The variables showing the largest effects on the state trajectory are marked with a star symbol.

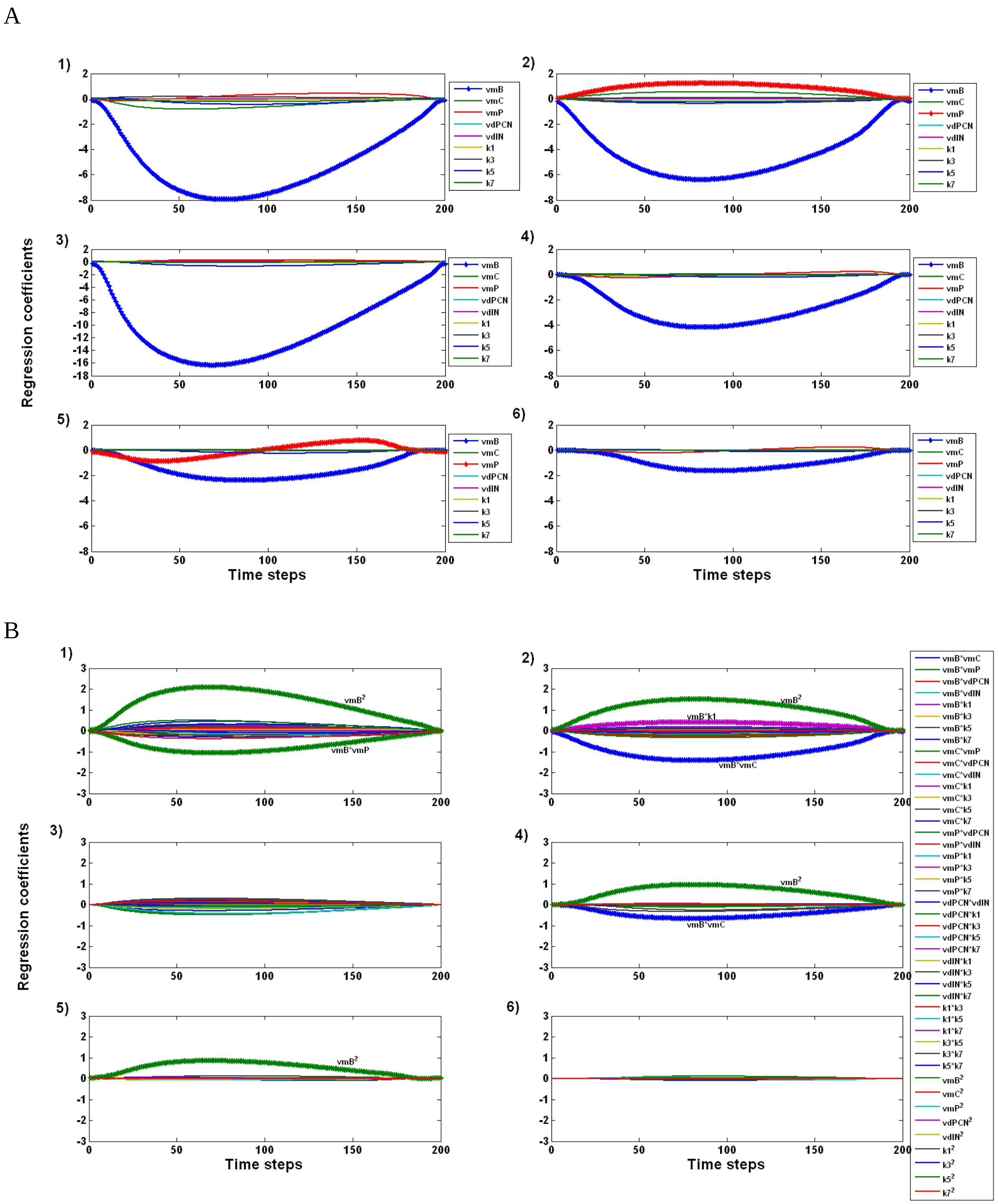

Figure S6. Results from the regional sensitivity analysis of the state variable B_N. Regression coefficients for A) the main effects of the circadian clock input parameters and B) the cross-terms and second order terms of the input parameters from the HC-PLSR-based sensitivity analysis of the state variable B_N. The variables showing the largest effects on the state trajectory are marked with a star symbol in panel A) and named in panel B). The six plots correspond to the same six clusters as used in the N-way HC-PLSR described in the main manuscript.

According to Figure S6, both the sensitivity of the state variable B_N to the circadian clock input parameters and the amount of interaction between the input parameters varied between the six clusters in the HC-PLSR. As was also seen in the N-way HC-PLSR, the state variable B_N seemed to have negligible sensitivity to the parameter k_7 in the parameter space analysed here, even though this parameter appeared in the differential equation for this state variable.

3.3 Separate sensitivity analysis for the state variable PC_{NP}

The global second order polynomial PLSR model obtained using the parameters and their cross-terms and second order terms as regressors and the logarithm of the PC_{NP} state trajectory (concentration of phosphorylated Per-Cry protein complex in the nucleus) as response gave a mean R^2-value over the trajectory of 0.92 in the test set prediction. There was no gain from using regional analysis of the input-output relationships for this state variable, and the regression coefficients for the 200 time steps in the trajectory from the global PLSR were therefore used as sensitivity measures (Figure S7). In the same way as the state variable I_N, PC_{NP} had to be logarithmised prior to the analysis, and this may explain why it could not be described in the N-way HC-PLSR metamodel including all state variables.

A

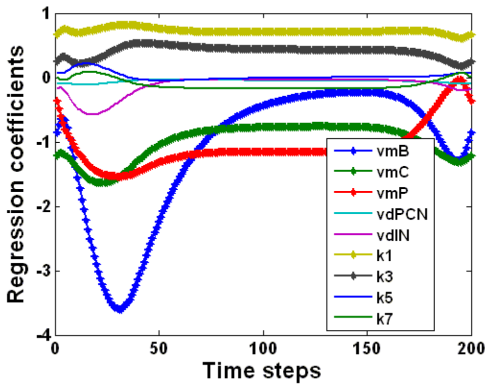

B

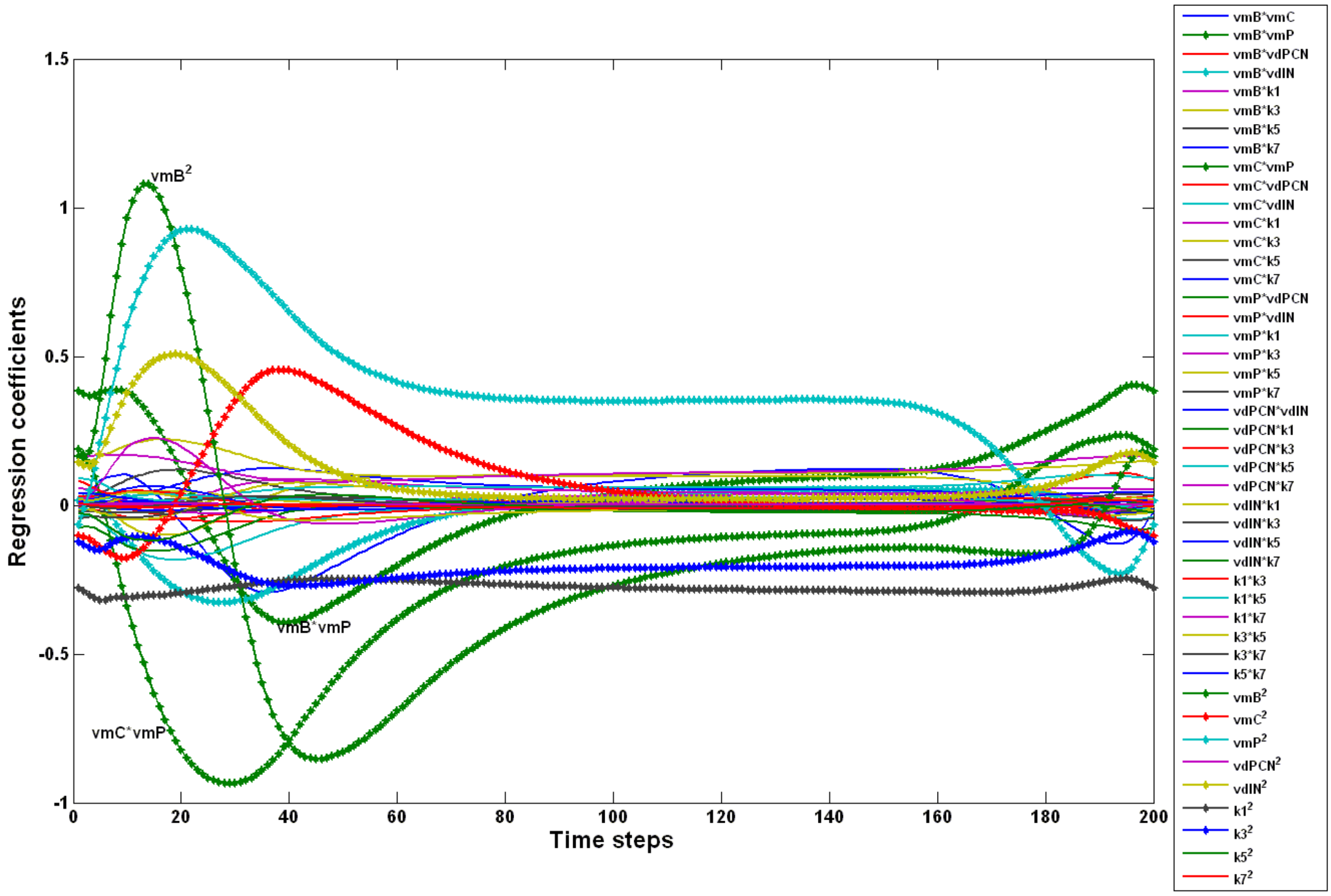

Figure S7. Results from the sensitivity analysis of the state variable *PC_{NP}*.
Regression coefficients for A) the main effects of the circadian clock input parameters and B) the cross-terms and second order terms of the input parameters from the global PLSR-based sensitivity analysis of the state variable PC_{NP}. The variables showing the largest effects on the state trajectory are marked with a star symbol.

As shown in Figure S7, the parameters having the largest effect on the PC_{NP} state trajectory were v_{mB}, v_{mC}, v_{mP}, k_1 and k_3. Several interactions between the input parameters were also identified, including an interaction between v_{mB} and v_{dIN}. However, like in the N-way HC-PLSR, no sensitivity to the parameter v_{dPCN} was detected, even though this parameter represented the rate of degradation of the phosphorylated Per-Cry complex in the nucleus (PC_{NP} represented the concentration of this protein in the nucleus).

3.4 Separate sensitivity analysis for the input parameter k_7

Additional simulations with the mammalian circadian clock model were carried out in order to explain the positive effect of k_7 on C_C seen in the NPLSR analysis in Cluster 1. All input parameters were kept constant at their mean values for Cluster 1, except k_7 (rate constant for the formation of the inactive Per-Cry-Clock-Bmal1 complex), which was varied at 8 different levels (Figure S8). The results from the additional simulations are shown in Figure S9.

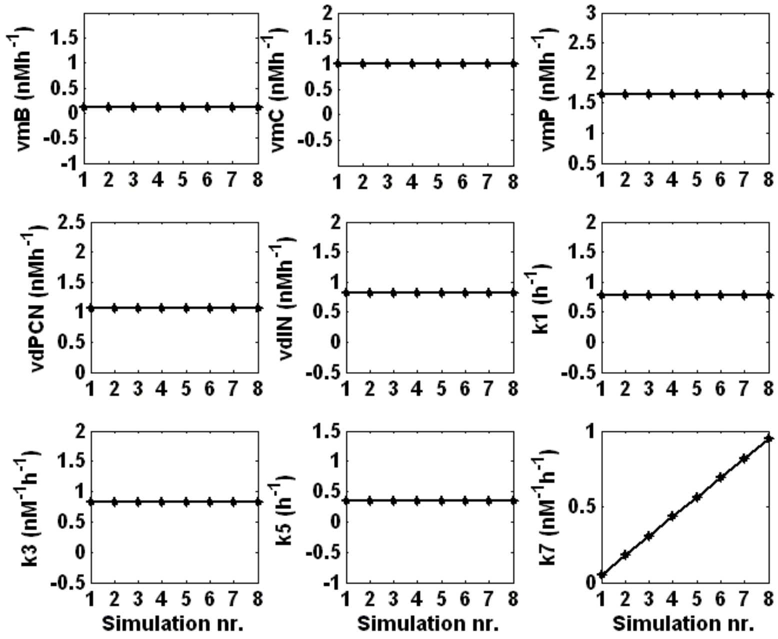

Figure S8. Parameter settings for additional simulations with the mammalian circadian clock model.

Parameter values for the eight new simulations are illustrated. The input parameter k_7 was varied at eight different levels, while the other parameters were kept at their average values from Cluster 1 (from the N-way HC-PLSR).

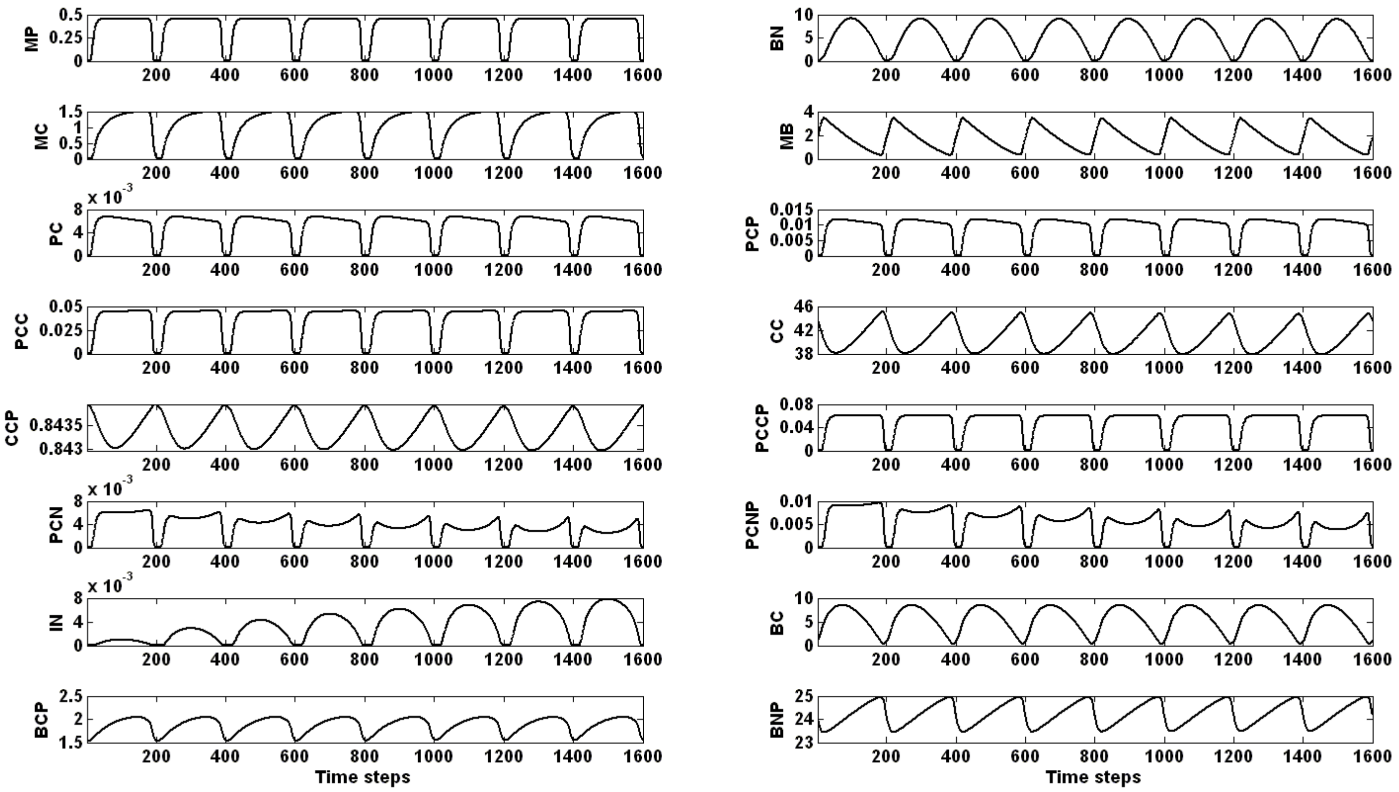

Figure S9. Results from the additional eight simulations with the mammalian circadian clock model.
Concatenated state variable trajectories for the eight new runs corresponding to the parameter values in Figure S8. All state variables are given in nM units.

The results above indicated that increasing k_7 resulted in a very small decrease in C_C and C_{CP}, a clear decrease in PC_N and PC_{NP} and an increase in I_N (which is very logical from the differential equation for I_N). Hence, this analysis did not confirm the positive effect of k_7 on C_C seen in the NPLSR analysis in Cluster 1. In order to analyse this input-output relationship further, a separate PLSR-based sensitivity analysis was carried out for the state variable C_C in Cluster 1 (explaining 94.6% of the cross-validated *Y*-variance). The results are shown in Figure S10.

A

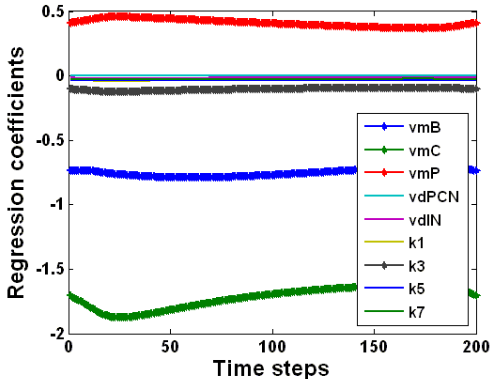

B

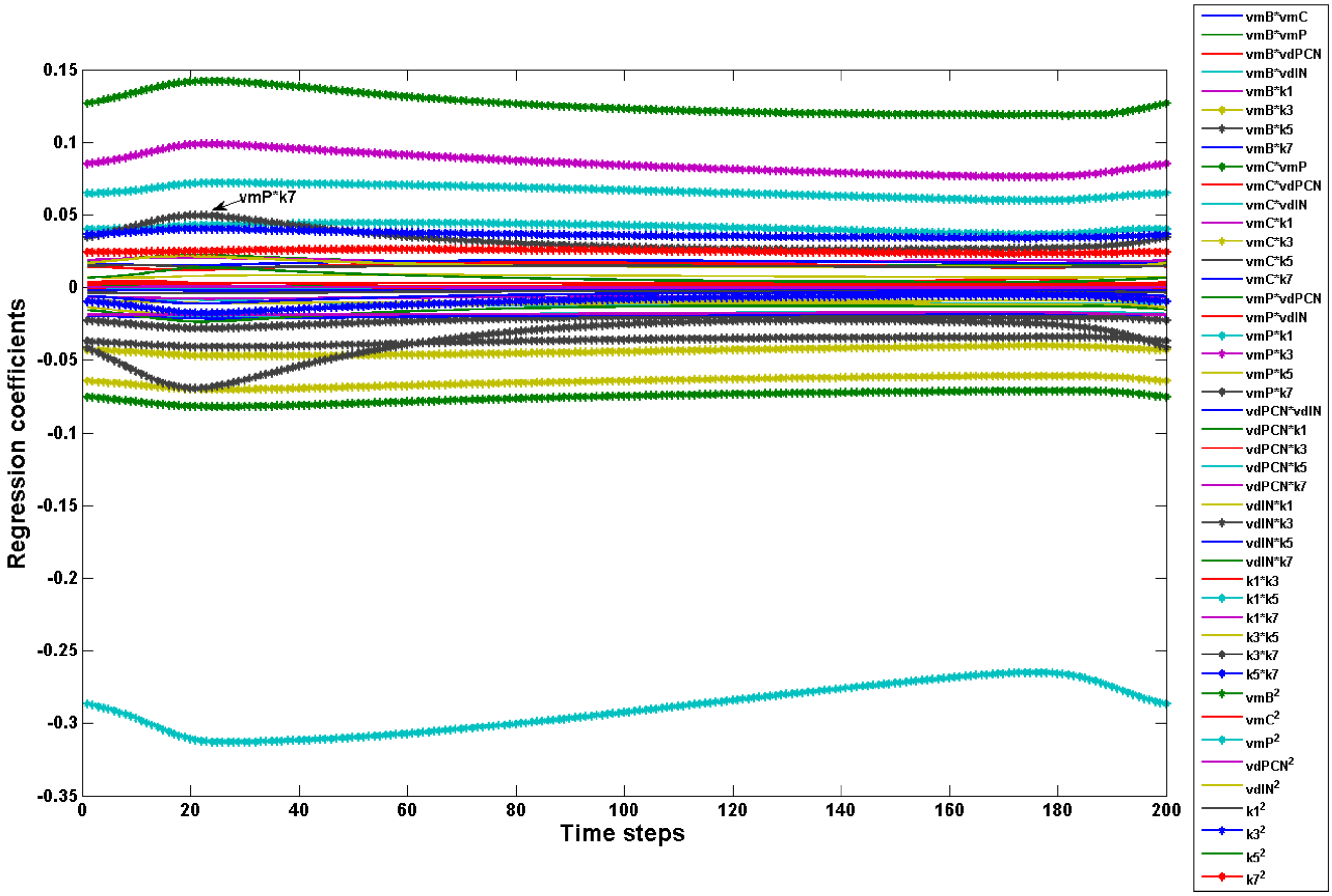

Figure S10. Results from the sensitivity analysis of the state variable C_C in Cluster 1. Regression coefficients for A) the main effects of the circadian clock input parameters and B) the cross-terms and second order terms of the input parameters from the PLSR-based sensitivity analysis of the state variable C_C in Cluster 1. The variables showing the largest effects on the state trajectory are marked with a star symbol.

Figure S10 showed that the effects of v_{mB}, v_{mC}, v_{mP} and k_3 on C_C indicated in the NPLSR analysis in the main manuscript were also manifested in the 2-way PLSR analysis of the input-output relationships in Cluster 1. However, the positive main effect of k_7 was not confirmed by the 2-way PLSR. However, several interaction terms involving k_7 seemed to have effects on C_C, such as the interaction between v_{mP} and k_7. Since cross-terms between the input parameters were not included in the N-way PLSR analysis, confounding of these interaction effects with the main effect of k_7 may explain the positive sensitivity to k_7 indicated by the N-way PLSR.

Supplementary material 4. Results from the method benchmarking

4.1 Results from inverse metamodelling using an unfolded state trajectory matrix

The state variable trajectory array was unfolded into a 2-way matrix by concatenating all state trajectories. The resulting matrix was used as regressor in a 2-way HC-PLSR to predict the circadian clock parameters (analogous to the inverse N-way HC-PLSR metamodelling). The clustering results from the 2-way HC-PLSR using the unfolded state trajectory matrix as regressor are given in Figure S11. The global parameter prediction accuracies were comparable to those obtained with the global inverse N-way metamodelling, but in the hierarchical metamodelling, the same predictive ability (on average) could not be achieved using 2-way HC-PLSR (data not shown). This was probably caused by the low quality of the clustering of the observations.

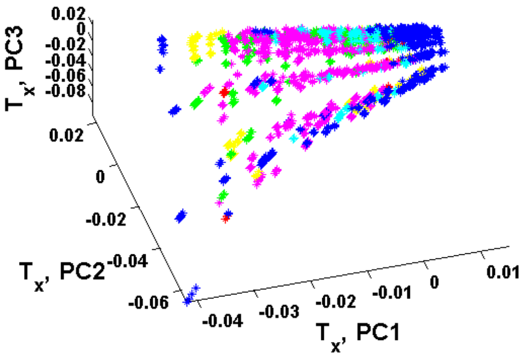

Figure S11. Clustering of the observations based on the unfolded state trajectory matrix. Plot of the *X*-scores (PC1-PC3) from the global inverse PLSR metamodelling using the unfolded state trajectory matrix as regressor and the parameters as response variables. The observations are coloured according to the cluster memberships used in the 2-way HC-PLSR. Cluster1=blue, cluster2=red, cluster3=yellow, cluster4=green, cluster5=magenta, cluster6=cyan. The clustering was done on the *X*-scores, using 12 PCs.

4.2 Results from inverse metamodelling using aggregated outputs derived from the state trajectories

From the state variable data, the following aggregated outputs were calculated: period of oscillation, time to convergence, bottom, peak, time to bottom and time to peak for each state variable trajectory. This resulted in 65 aggregated outputs (see Figure S12). The parameters of the mammalian circadian clock model were predicted from these aggregated outputs using (2-way) HC-PLSR. This resembles the approach presented by Sarkar and Sobie [1], except they used conventional PLSR instead of HC-PLSR, and in [1] an inversion of the regression coefficient matrix derived by predicting phenotypes from parameters was used instead of calibrating a PLSR model directly using the parameters as responses like we did here. The clustering results from the 2-way HC-PLSR using aggregated outputs to predict the circadian clock parameters are given in Figure S13. As when using the unfolded state trajectory matrix as regressor, the global parameter prediction accuracies were also here comparable to those obtained with the global inverse N-way metamodelling, but in the hierarchical metamodelling, the same predictive ability (on average) could not be achieved using 2-way HC-PLSR (data not shown).

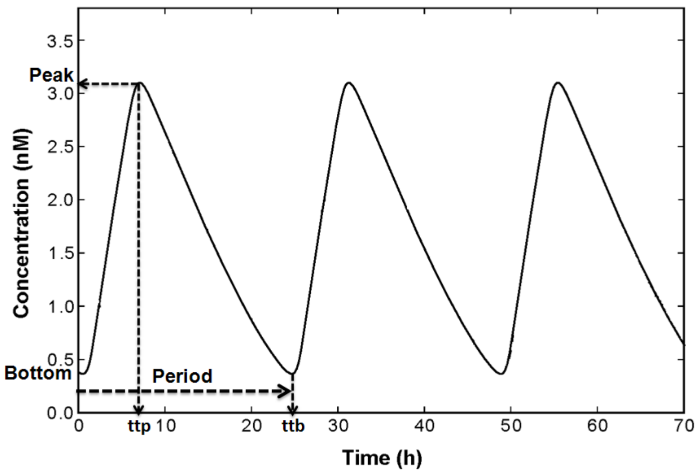

Figure S12. Aggregated outputs derived from the mammalian circadian clock state trajectories.
Illustration of the calculated aggregated outputs of one of the 16 circadian clock state variables, M_B. ttb=time to bottom, ttp=time to peak.

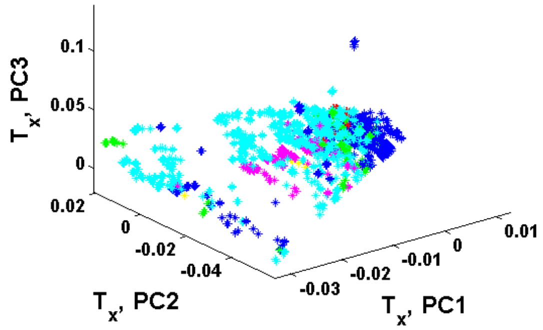

Figure S13. Clustering of the observations based on the aggregated outputs.
Plot of the *X*-scores (PC1-PC3) from the global inverse PLSR metamodelling using the aggregated outputs as regressors and the parameters as response variables. The observations are coloured according to the cluster memberships used in the 2-way HC-PLSR. Cluster1=blue, cluster2=red, cluster3=yellow, cluster4=green, cluster5=magenta, cluster6=cyan. The clustering was done on the *X*-scores, using 12 PCs.

4.3 Conclusions from method benchmarking

As shown in Figure S11 and S13, the calibration set observations were not clustered into well-defined distinctly separated clusters when the clustering was based on PLSR scores from a global PLSR model using the unfolded state trajectory matrix or the aggregated outputs as regressors to predict the circadian clock parameters. As shown in the main manuscript, the clustering based on the NPLSR factors derived from the 3-way state trajectory array resulted in considerably more well-defined clusters. Hence, using the entire time series in NPLSR gave a more distinct and presumably more biologically relevant separation of the observations into clusters within which to carry out regional modelling, and was the most suitable method for exploration of the input-output mapping.

The global parameter prediction accuracies from the 2-way metamodelling were comparable to those obtained with the global inverse N-way metamodelling. However, in the hierarchical metamodelling, neither the unfolded state trajectories nor the aggregated outputs could predict the parameters with as high accuracy (on average) using 2-way HC-PLSR as with the N-way HC-PLSR

due to a lower quality of the clustering. Hence, there is a clear gain of using multi-way methodology.

4.4 References

1. Sarkar AX, Sobie EA: **Regression Analysis for Constraining Free Parameters in Electrophysiological Models of Cardiac Cells**. *PLoS Comput Biol* 2010, **6**.
